# A Trisbenzimidazole Phosphoramidite Building Block Enables High-Yielding Syntheses of RNA-Cleaving Oligonucleotide Conjugates

**DOI:** 10.3390/molecules25081842

**Published:** 2020-04-16

**Authors:** Felix Zellmann, Michael W. Göbel

**Affiliations:** Institute of Organic Chemistry and Chemical Biology, Goethe University Frankfurt, Max-von-Laue-Str. 7, D-60438 Frankfurt am Main, Germany; Felix.Zellmann@gmx.net

**Keywords:** 2-aminobenzimidazole, artificial ribonuclease, DNA-LNA mixmers, guanidine analogs

## Abstract

The RNA cleaving catalyst tris(2-aminobenzimidazole) when attached to the 5’ terminus of oligonucleotides cuts complementary RNA strands in a highly site-specific manner. Conjugation was previously achieved by the acylation of an amino linker by an active ester of the catalyst. However, this procedure was low yielding and not reliable. Here, a phosphoramidite building block is described that can be coupled to oligonucleotides by manual solid phase synthesis in total yields around 85%. Based on this chemistry, we have now studied the impact of LNA (locked nucleic acids) nucleotides on the rates and the site-specificities of RNA cleaving conjugates. The highest reaction rates and the most precise cuts can be expected when the catalyst is attached to a strong 5’ closing base pair and when the oligonucleotide contains several LNA units that are equally distributed in the strand. However, when placed in the 5’ position, LNA building blocks tend to diminish the specificity of RNA cleavage.

## 1. Introduction

The interest in artificial ribonucleases was triggered some decades ago by the early work on antisense therapeutics. Structurally modified oligonucleotides were developed in these years in order to achieve sufficient levels of RNA affinity and stability against enzymatic degradation. Hybrids of heavily modified strands and their target RNAs, on the other hand, often lost their ability to recruit RNAse H [1,2]. Synthetic RNA cleavers were designed and attached to oligonucleotides to render their activity sequence-specific to compensate this disadvantage [3,4,5,6,7]. Since then, complexes of lanthanide and of other cations have been most often used for this purpose [8,9,10,11,12,13]. Although in modern life sciences gapmers [2,14,15] and siRNAs [16] are the preferred tools for achieving the site-specific cleavage of RNA strands, there is still interest in synthetic ribonucleases and recent years have seen important progress in the field. Effective catalysts that are based on metal ions [17,18,19,20,21,22,23], guanidines or polyamines [24,25,26,27], peptide conjugates [28,29,30,31,32], and deoxyribozymes [33,34] have been described. Starting from heterocyclic guanidine analogs [35], we have investigated the conjugates of tris(2-aminobenzimidazoles) and different types of oligonucleotides, such as DNA [36], PNA [37,38], or DNA-LNA mixmers [39], which were shown to act as sequence-specific metal-free RNA cleavers. Product inhibition prevented multiple turnover when the catalyst was attached to a terminal position of the conjugates and the reaction was run under isothermal conditions, because the number of base pairs in the conjugate-substrate hybrid does not change in the cleavage step [36]. However, this problem could be resolved by placing the catalyst in a central position. Such conjugates were shown to act with multiple substrate turnover [38]. 

**Figure 1:**
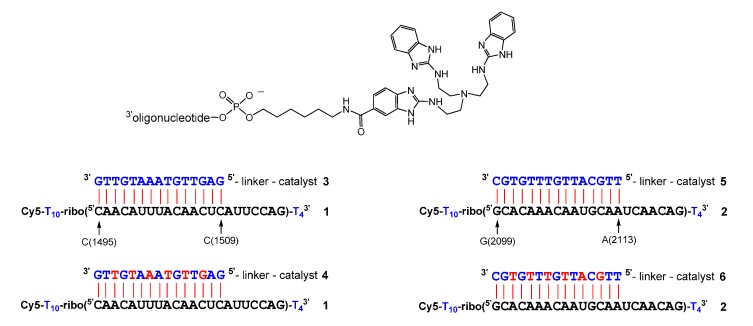
Sequence of RNAs **1** and **2** and of conjugates **3**–**6**. DNA and RNA nucleotides are depicted in blue and black, LNA nucleotides in red. The general structure of the conjugates is shown on top.

**Scheme 1:**
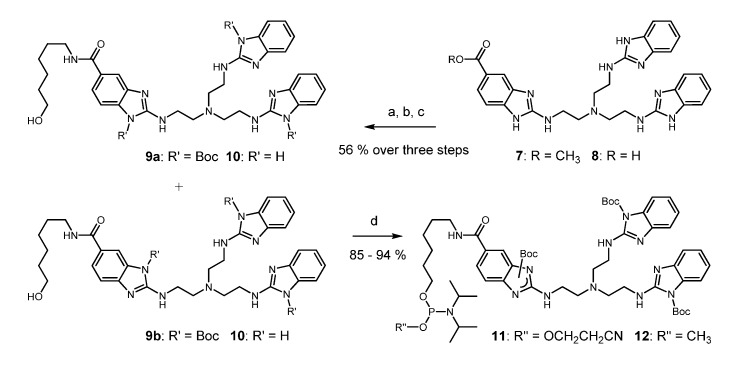
Syntheses of tris(2-aminobenzimidazole) phosphoramidites **11** and **12**. Conditions: (**a**): 6 M HCl_(aq)_, reflux, 6 h; (**b**): Boc_2_O, DIPEA, DMF, rt, 3 h; (**c**): 6-aminohexan-1-ol, DIC, HOBt, DMF, rt, 16 h; (**d**): *N,N*-diisopropylcyanoethylphosphonamidic chloride or *N,N*-diisopropylmethylphosphon-amidic chloride, NEt_3_, CH_2_Cl_2_, 0 °C to rt, 45 min.

In a recent study [39], the 15mer DNA conjugates **3** and **5** were synthesized and then used to cleave 22mer model RNAs **1** and **2**, derived from the *PIM1* 3’-UTR (Figure 1). The sequences of RNAs **1** and **2** correspond to a validated siRNA cleavage site (nt 1495–1509) and a binding site for miR-33a (nt 2099–2113) [40]. When 150 nM of RNA **1** were incubated with increasing amounts of conjugate **3**, concentrations of 3 µM were required to obtain rate saturation (50 mM Tris buffer, pH 8.0, 37 °C). A substrate half-life of 14 h was observed under such conditions and a value of 15 h for the cleavage of RNA **2** by conjugate **5**. We then replaced the 15mer DNA part by DNA-LNA mixmers to improve substrate affinities (Figure 1). As expected, rate saturation with conjugates **4** and **6** was now observed at lower concentrations of 750 nM. To our surprise, the incorporation of LNA building blocks also had a major impact on kinetics. The half-life of RNA **2**, when incubated with conjugate **6**, dropped from 15 h to 10 h. The half-life of RNA **1** even dropped to 3.5 h in the presence of mixmer conjugate **4**. Apart from cleaving the 22mer model RNA **1**, this conjugate was also shown to precisely cut *PIM1* derived 155mer and 412mer transcripts [39]. In the present study, we investigated the influence of LNA nucleotides on cleavage patterns and kinetics in a more systematic way. However, our previous method of catalyst attachment was not fully satisfying: a tritylated 5’ amino linker was deprotected, while the oligonucleotide strand was still bound to the support. Catalyst **8** (Scheme 1), activated with DIC and HOBt, was then added and it formed a stable amide bond. The conjugation yield did not exceed 30% and could occasionally drop to zero due to accidental acylation of the amino linker by capping reagents or by protecting group migration. Here, we describe two phosphoramidite building blocks of the catalyst (Scheme 1, compounds **11** and **12**) that can be manually attached to the 5’ end of DNA or DNA-LNA mixmers in excellent yield.

## 2. Results

2-Aminobenzimidazoles are nucleophilic species that must be protected before phosphitylation by an electron withdrawing group. Starting from the readily accessible ester **7** [39], acid induced hydrolysis (**8**) and reaction with di-*tert*-butyl dicarbonate converted the compound into a triply acylated intermediate that was subsequently coupled with 6-aminohexanol. Steps a–c could be performed in a one-pot procedure. A mixture of two constitutional isomers **9a** and **9b** was isolated in 56% yield and in batches of 11.5 g. Boc residues are normally stable against nucleophilic attack. However, in the present case, standard conditions of oligonucleotide deprotection (aqueous ammonia/EtOH 3: 1, 55 °C, 16 h) completely removed all Boc groups from **9a** and **9b** producing compound **10** without any visible degradation of the trisbenzimidazole (for details, see Supplementary Materials). Furthermore, compounds **9a** and **9b** were fully compatible with typical reagents for oligonucleotide synthesis, such as 0.5 M 5-ethylthio-1*H*-tetrazole (ETT) in acetonitrile and commercial oxidizer solution. Before testing Boc as protecting group, we experimented with acetyl, pivaloyl, and ethoxycarbonyl. With the high reactivity of *N*-acyl imidazoles in mind, it was not unexpected to find all of these groups insufficiently stable. In the last step, the phosphitylation of alcohols **9a** and **9b** gave access to the phosphoramidites **11** and **12** (each consisting of two constitutional isomers) in good to excellent yields (Scheme 1). However, the cyanoethoxy amidite **11** showed significant formation of *H*-phosphonate during evaporation of the solvent at 30 °C after column chromatography and also after storage at −20 °C for a couple of days. Although the reason is not yet clear, decomposition might result from some residual Brønsted basicity of the protected trisbenzimidazole. The methoxy amidite **12**, in contrast, was stable and it could be stored for several months. Manual coupling to the 5’ end of resin-bound oligonucleotides and standard deprotection resulted in excellent total yields of conjugates around 85% (determined by HPLC). Samples of amidite **11** containing some *H*-phosphonate, nevertheless coupled with comparable efficiency. Unfortunately, we failed to transfer this reaction to an automated DNA synthesizer under standard conditions when amidite and tetrazole are pre-mixed and then pumped into the reaction column (PerSeptive Expedite, 1.0 μmol scale). It seems that after activation the phosphoramidite rapidly undergoes some unknown side reactions. By increasing the pump rate, coupling yields up to 60% could be obtained, but they never reached the almost quantitative conversion that was seen in the manual method. Thus, conjugates **13**–**20** were prepared by the manual conjugation protocol, in which phosphoramidite activation directly occurs on the solid support.

**Figure 2:**
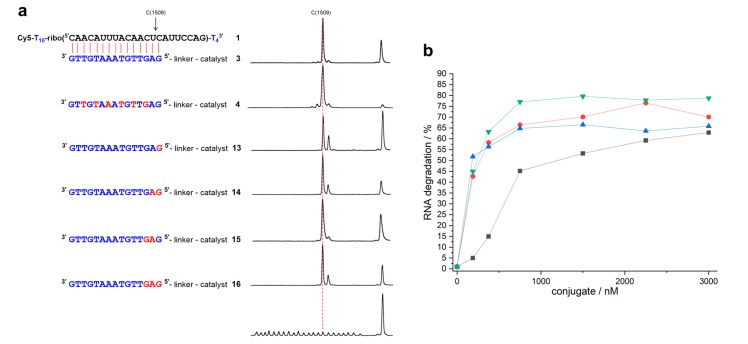
(**a**) Structures of RNA **1** and complementary mixmer conjugates **13**–**16** with their corresponding cleavage patterns and hydrolysis ladder of RNA **1**. DNA, LNA, and RNA nucleotides are shown in blue, red, and black letters, respectively. The red dashed line indicates position 15, the last nucleotide in the RNA-conjugate duplex (150 nM of RNA **1**, 3 µM of conjugate, 50 mM Tris buffer (pH 8.0), 37 °C, 19–21 h). (**b**) Percentage of RNA cleavage after constant time as a function of conjugate concentration (150 nM of RNA **1**, 50 mM Tris buffer (pH 8.0), 37 °C, 20 h). Black squares: conjugate **13**. Red circles: conjugate **14**. Blue triangles: conjugate **15**. Green triangles: conjugate **16**. Data points are connected by lines for the sake of clarity.

The 22mer RNA parts of substrates **1** and **2** are both embedded into a chain of 14 thymidine residues. The strands are labeled with a fluorescent dye allowing for us to detect them and their cleavage fragments in an ALFexpress sequencer. Upon base induced fragmentation, only linkages between ribonucleotides can be cleaved. Thus, 22 well resolved peaks became visible in the hydrolysis ladder. The 3’ terminal thymidine tetramer helped to separate the peaks of the longest fragment from the substrate peak (Figure 2a). When RNA **1** was cleaved by conjugate **3**, a comparison of the cleavage pattern and the base ladder immediately showed that the reaction mainly occurred between nucleotides 15 and 16, directly at the end of the duplex. The mixmer conjugate **4** induced a very similar pattern (Figure 2a). However, when the 5’ terminal G was replaced by an LNA analog, additional cleavage between nucleotides 16 and 17 increased. This effect was visible for conjugates **13**, **14**, and **16**, independent from the presence of further LNA building blocks in direct proximity. In contrast, conjugate **15** with a 5’ terminal deoxynucleotide fairly reproduced the cleavage pattern of conjugates **3** and **4**.

**Figure 3:**
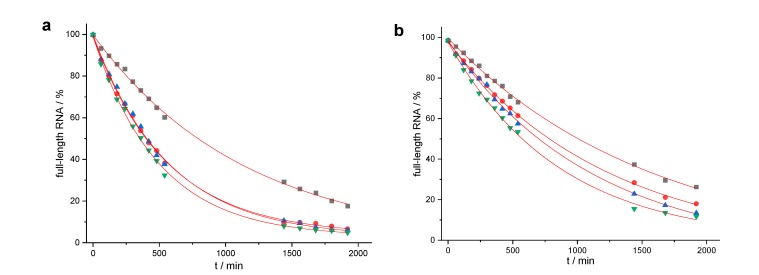
Determination of rate constants: (**a**) 150 nM of RNA **1** were incubated with 3 µM of **13**, **14, 15** or **16** (50 mM Tris buffer (pH 8.0), 37 °C); (**b**) 150 nM of RNA **2** were incubated with 3 µM of **17**, **18**, **19,** or **20** (50 mM Tris buffer (pH 8.0), 37 °C) The amount of residual full-length RNA is shown as a function of time. Representative examples of single experiments are shown, and the data were fitted to a first-order rate model (single exponential decay). Mean values of *k*_max_ (calculated from three independent experiments) were accurate within ± 15%. (**a**) Reaction of RNA **1** with conjugates **13** (black squares), **14** (red circles), **15** (blue triangles), **16** (green triangles). (**b**) Reaction of RNA **2** with conjugates **17** (black squares), and **18** (red circles), **19** (blue triangles), **20** (green triangles).

In rate saturation experiments (Figure 2b), conjugate **13** showed weaker substrate binding as compared to compounds **14**–**16** containing two or more LNA nucleotides. Cleavage kinetics for compound **13** was determined at concentrations of 3 µM, as in the case of the DNA conjugate **3**. For conjugates **14**–**16**, rate saturation, indicating full occupation of the substrate strands, was already attained at concentrations of 750 nM. Nevertheless, we determined the RNA cleavage kinetics for all new conjugates at concentrations of 3 µM (50 mM Tris buffer, pH 8.0, 37 °C). In all cases, the data could be fitted to a first-order rate model (Figure 3).

The single LNA nucleotide that was present in compound **13** did not significantly change reaction rates (*k*_max_ = 0.052 h^−1^, *t*_1/2_ = 13.3 h) when compared to the unmodified DNA conjugate **3** (*k*_max_ = 0.050 h^−1^, *t*_1/2_ = 14 h), but downgraded the precision of the cut. Conjugates containing two or three LNA nucleotides reacted faster: **15** (*k*_max_ = 0.107 h^−1^, *t*_1/2_ = 6.5 h) ≈ **14** (*k*_max_ = 0.108 h^−1^, *t*_1/2_ = 6.4 h) < **16** (*k*_max_ = 0.124 h^−1^, *t*_1/2_ = 5.6 h) (Figure 3a). However, none of the new conjugates could compete with the LNA-rich conjugate **4** (*k*_max_ = 0.20 h^−1^, *t*_1/2_ = 3.5 h). Although the introduction of LNA nucleotides increased the substrate affinities and reaction rates in general, they induced cleavage in the n+1 position when brought to the 5’ terminus.

**Figure 4:**
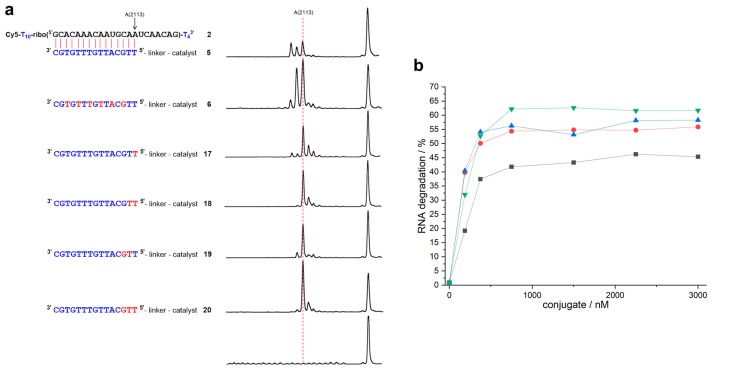
(**a**) Structures of RNA **2** and of complementary mixmer conjugates **17**–**20** with their corresponding cleavage patterns. DNA, LNA, and RNA nucleotides are shown in blue, red, and black letters, respectively. The red dashed line indicates position 15, the last nucleotide in the RNA-conjugate duplex (150 nM of RNA **2**, 3 µM of conjugate, 50 mM Tris buffer (pH 8.0), 37 °C, 19–21 h). (**b**) Percentage of RNA cleavage after constant time as a function of conjugate concentration (150 nM of RNA **2**, 50 mM Tris buffer (pH 8.0), 37 °C, 20 h). Black squares: conjugate **17**. Red circles: conjugate **18**. Blue triangles: conjugate **19**. Green triangles: conjugate **20**. Data points are connected by lines for the sake of clarity.

The duplex that formed from RNA **2** and conjugate **5** differs from the previous cases in so far as the two closing base pairs are weak and prone to fraying: In addition to cleavage between nucleotides 15 and 16, the reaction also occurs after nucleotides 13 and 14 (Figure 4a). In conjugate **17** with a single 5’ terminal LNA nucleotide, fraying is reduced, but not fully suppressed. Furthermore, additional cleavage occurs in positions n + 1 (and n + 2), as in the case of conjugate **13**. When compared to the DNA conjugate **5** (*k*_max_ = 0.046 h^−1^, *t*_1/2_ = 15 h), the LNA building block even retards RNA cleavage (*k*_max_ = 0.038 h^−1^, *t*_1/2_ = 18.3 h). Site-selectivity improves in the presence of two LNA building blocks, but, depending on the location, either residual fraying or some additional cleavage in position n + 1 occurs. The reaction rates of conjugates **18** (*k*_max_ = 0.049 h^−1^, *t*_1/2_ = 14.2 h) and **19** (*k*_max_ = 0.053 h^−1^, *t*_1/2_ = 13.0 h) come close to the value of the DNA conjugate **5**. Conjugate **20** (*k*_max_ = 0.071 h^−1^, *t*_1/2_ = 9.8 h) with three 5’ terminal LNA nucleotides not only cleaves as fast as the DNA-LNA mixmer **6** (*k*_max_ = 0.069 h^−1^, *t*_1/2_ = 10 h), it also shows the best site-selectivity that was observed with RNA **2**: More than 70% of the strands are cleaved directly after the duplex region (Table 1). 

## 3. Discussion

The availability of the phosphoramidite building blocks **11** and **12** has greatly facilitated the covalent attachment of oligonucleotides and RNA cleaving trisbenzimidazoles. This approach has allowed us to investigate the impact of LNA nucleotides in a more systematic way. RNA **1** represents the first type of target where cleavage is intended to occur after a strong G-C closing base pair that prevents the fraying of the substrate-conjugate duplex. The highest level of site-specificity is already seen with the unmodified DNA conjugate **3**. 89% of the fragments are directly cleaved after the final base pair (Table 1). The introduction of LNA building blocks does not further improve this number. On the contrary, LNA as part of the closing base pair leads to 20–30% cleavage in the n + 1 position. Interestingly, it has been shown in a previous study that duplex stabilization by LNA nucleotides is much less pronounced when brought to the 5’ terminus instead to internal positions [41]. The incorporation of several LNA modifications, although it does not improve the precision of the cut, can accelerate the reaction up to four-fold. The situation is different when the closing base pairs of the RNA-conjugate duplex are weak as in the case of RNA **2**. LNA building blocks have a minor impact on cleavage rates, but they considerably prevent fraying and improve site-specificity (Table 1). However, the cleavage rates and specificities both stay behind the numbers found for RNA **1**. From these observations, some general conclusions for the design of synthetic RNA cleavers can be drawn: In all conjugates tested so far, the trisbenzimidazole catalyst works best when placed after a 5’ terminal G-C base pair. The stacking of the catalyst on the purine nucleobase might be important. Several LNA modifications evenly distributed in the DNA part of the conjugate can largely increase reaction rates. LNA in the 5’ position, however, is contraproductive and it spoils the site-specificity of the conjugate. The reason, steric hindrance or conformational effects, is not clear at present. The high stability of DNA-LNA mixmers, such as **4** and **6**, and their independence from metal ions may allow for them not only to act in buffer solution, but also in living cells. 

## 4. Materials and Methods 

### 4.1. General

All of the chemicals were reagent grade and used as purchased. All of the reactions were performed under an argon atmosphere. The reactions were monitored by TLC using silica gel 60 F-254 aluminum sheets (Merck, Darmstadt, Germany). Compounds were visualized by UV light (254 and 366 nm). Column chromatography was carried out on silica gel 60 (0.04–0.063 mm). Proton nuclear magnetic resonance (^1^H-NMR) spectra and carbon nuclear magnetic resonance (^13^C-NMR) were recorded at 300 K with *Bruker* AV 300 (^1^H: 300 MHz; ^13^C: 75.5 MHz) and *Bruker* AV 500 (^1^H: 500 MHz; ^13^C: 125.8 MHz) NMR spectrometers (Bruker, Karlsruhe, Germany). Chemical shifts for protons are reported in parts per million (δ scale) and internally referenced to the proton resonances of the solvent (C*D*Cl_3_: δ 7.26, DMSO-*d*_6_: δ 2.50). The chemical shifts for carbon are reported in parts per million (δ scale) and referenced to the carbon resonances of the solvent (C*D*Cl_3_: δ 77.16, DMSO-*d*_6_: δ 39.52). The data are represented, as follows: chemical shift, multiplicity (s = singlet, bs = broad singlet, d = doublet, t = triplet, q = quartet, quin = quintet, m = multiplet, dd = doublet of doublets, td = triplet of doublets), coupling constants in Hz, and integration. The IR spectra were recorded with a Jasco FT/IR-420 spectrometer (Jasco Germany GmbH, Pfungstadt, Germany) equipped with an ATR unit. ESI-MS spectra were obtained on a Fisons VG Plattform II (Fisons VG Biotech MS, Altrincham, UK). HRMS spectra were recorded on a MALDI LTQ Orbitrap mass spectrometer from Thermo Scientific (Waltham, MA, USA).

*Di-tert-butyl-2,2’-((((2-((1-(tert-butoxycarbonyl)-6-((6-hydroxyhexyl)carbamoyl)-1H-benzo[d]imidazol-2-yl)amino)ethyl)azanediyl)bis(ethane-2,1-diyl))bis(azanediyl))bis(1H-benzo[d]imidazole-1-carboxylate)* (**9a/b**): A suspension of the ester **7** [39] (12.4 g, 22.4 mmol) in 6 M hydrochloric acid (150 mL) was refluxed for 6 h. The solvent was removed under reduced pressure and the residue was dried *in vacuo* to obtain a light brown solid. To a solution of the carboxylic acid **8** and DIPEA (13.7 mL, 78.5 mmol, 3.5 eq) in DMF (250 mL) Boc_2_O (17.1 g, 78.5 mmol, 3.5 eq) was added. After 3 h at room temperature, HOBt (6.87 g, 44.9 mmol, 2 eq), DIC (5.68 g, 44.9 mmol, 2 eq), and 6-aminohexan-1-ol (5.26 g, 44.9 mmol, 2 eq) were added and the solution was stirred at room temperature over night. The solvent was removed under reduced pressure and the residue was purified via column chromatography (SiO_2_, DCM/MeOH 20:1) to obtain the mixture of **9a** and **9b** as a light yellow foam. Yield: 11.7 g (12.5 mmol, 56% over three steps). R_f_ = 0.37, 0.42 (DCM/MeOH 15:1). ^1^H NMR (500 MHz, C*D*Cl_3_, mixture of two isomers): *δ* [ppm] = 8.12 (d, *J* = 1.8 Hz, 0.6 H), 7.85 (t, *J* = 5.2 Hz, 0.6 H), 7.76–7.72 (m, 2.9 H), 7.56 (dd, *J* = 8.4 Hz, *J* = 1.7 Hz, 0.6 H), 7.51–7.48 (m, 2.9 H), 7.33 (dd, *J* = 7.9 Hz, *J* = 1.2 Hz, 2 H), 7.26 (d, *J* = 8.1 Hz, 0.6 H), 7.13 (td, *J* = 7.7 Hz, *J* = 1.2 Hz, 2 H), 6.97 (tt, *J* = 7.6 Hz, *J* = 1.4 Hz, 2 H), 6.56 (t, *J* = 5.8 Hz, 0.4 H), 6.34 (t, *J* = 5.8 Hz, 0.6 H), 3.74–3.58 (m, 6 H), 3.60 (q, *J* = 6.2 Hz, 2 H), 3.42 (q, *J* = 6.7 Hz, 2 H), 3.01 (q, *J* = 5.2 Hz, 2 H), 2.96 (t, *J* = 5.3 Hz, 4 H), 1.61–1.59 (m, 2 H), 1.56–1.52 (m, 2 H), 1.49 (s, 5 H), 1.48 (s, 3.7 H), 1.46 (s, 18 H), 1.40–1.35 (m, 4 H). ^13^C NMR (126 MHz, C*D*Cl_3_, mixture of two isomers): *δ* [ppm] = 167.83, 167.80, 155.7, 155.9, 154.2, 154.1, 151.1, 151.0, 150.8, 150.7, 145.8, 142.5, 142.32, 142.26, 132.8, 131.0, 130.9, 130.52, 130.47, 126.6, 124.39, 124.36, 122.7, 120.50, 120.47, 120.4, 116.26, 116.22, 115.4, 114.2, 114.1, 113.92, 113.90, 113.7, 86.29, 86.26, 85.7, 85.6, 62.5, 62.4, 54.82, 54.76, 54.72, 54.6, 41.7, 41.6, 41.5, 40.0, 39.9, 32.64, 32.57, 29.82, 29.75, 29.6, 28.2, 27.91, 27.87, 26.7, 26.6, 25.43, 25.39. IR: *ṽ* [cm^−1^] = 3379 (w), 2976 (w), 2929 (w), 2856 (w), 1720 (s), 1626 (m), 1601 (m), 1574 (s), 1502 (w), 1456 (m), 1433 (w), 1394 (w), 1367 (m), 1352 (m), 1333 (s), 1296 (m), 1281 (m), 1261 (m), 1213 (m), 1334 (s), 1084 (w), 1055 (w), 1018 (w), 922 (w), 904 (w), 866 (w), 833 (m), 768 (m), 756 (m), 739 (s). MS (ESI+): *m*/*z* (%) = 938.73 (100) [M + H]^+^, calcd for C_49_H_68_N_11_O_8_: 938.52. HRMS (MALDI): *m*/*z* = 938.52458 [M + H]^+^, calcd for C_49_H_67_N_11_O_8_ + H^+^: 938.52468.

*2-((2-(Bis(2-((1H-benzo[d]imidazol-2-yl)amino)ethyl)amino)ethyl)amino)-N-(6-hydroxy-hexyl)-1H-benzo[d]imidazole-5-carboxamide* (**10**). To a solution of the carboxylic acid **8** (1.00 g, 1.54 mmol, 1 eq) and DIPEA (1.08 mL, 6.17 mmol, 4 eq) in DMF (3 mL) HOBt (0.21 g, 1.54 mmol, 1 eq) and DIC (0.19 g, 1.54 mmol, 1 eq) was added. After 1 h at room temperature, a solution of 6-aminohexan-1-ol (0.18 g, 1.54 mmol, 1 eq) in DMF (1 mL) was added dropwise. After stirring at room temperature overnight, the solvent was removed under reduced pressure and the residue was purified via column chromatography (SiO_2_, DCM/MeOH 10:1 + 1% NEt_3_) to obtain the product as a light yellow foam. Yield: 0.45 g (0.71 mmol, 46%). R_f_ = 0.33 (DCM/MeOH 10:1 + 1% NEt_3_). ^1^H NMR (300 MHz, DMSO-*d*_6_): *δ* [ppm] = 8.16 (t, *J* = 5.7 Hz, 1 H), 7.64 (d, *J* = 1.6 Hz, 1 H), 7.44 (dd, *J* = 8.3 Hz, *J* = 1.7 Hz, 1 H), 7.14–7.08 (m, 5 H), 6.88–6.82 (m, 4 H), 6.81 (bs, 1 H), 6.68 (bs, 2 H), 3.44–3.35 (m, 8 H), 3.22 (q, *J* = 6.6 Hz, 2 H), 2.78 (t, *J* = 6.5 Hz, 6 H), 1.50 (quin, *J* = 6.6 Hz, 2 H), 1.41 (quin, *J* = 6.4 Hz, 2 H), 1.32–1.27 (m, 4 H). ^13^C NMR (75 MHz, DMSO-*d*_6_): *δ* [ppm] = 172.5, 167.0, 156.9, 155.6, 125.8, 119.2, 111.6, 110.3, 60.7, 57.9, 53.7, 40.6, 39.2, 32.5, 29.4, 26.5, 25.3, 21.4. MS (ESI+): *m*/*z* (%) = 638.45 (100) [M + H]^+^, calcd for C_34_H_44_N_11_O_2_: 638.37. HRMS (MALDI): *m/z* = 638.36654 [M + H]^+^, calcd for C_34_H_43_N_11_O_2_ + H^+^: 638.36740.

*Di-tert-butyl-2,2’-((((2-((1-(tert-butoxycarbonyl)-6-((6-(((2-cyanoethoxy)(diisopropylamino)-phosphino)oxy)hexyl)carbamoyl)-1H-benzo[d]imidazol-2-yl)amino)ethyl)azanediyl)bis-(ethane-2,1-diyl))bis(azanediyl))bis(1H-benzo[d]imidazole-1-carboxylate)* (**11**). To a solution of the alcohols **9a/b** (1.0 g, 1.07 mmol, 1 eq) and NEt_3_ (0.74 mL, 5.33 mmol, 5 eq) in dry DCM (10 mL) *N,N*-diisopropylcyanoethylphosphonamidic chloride (0.50 g, 2.13 mmol, 2 eq) was added at 0 °C. After 15 min. at 0 °C and 30 min. at room temperature, saturated NaHCO_3_ solution was added. The phases were separated and the aqueous layer was extracted with DCM. The extracts were dried over MgSO_4_, the solvent was removed under reduced pressure at 30 °C, and the residue was purified via column chromatography (SiO_2_, EtOAc/MeOH 25:1 + 2% NEt_3_). Yield: 1.03 g (0.905 mmol, 85%). R_f_ = 0.27, 0.32 (EtOAc/MeOH 25:1 + 2% NEt_3_). ^1^H NMR (300 MHz, DMSO-*d*_6_, mixture of two isomers): *δ* [ppm] = 8.29 (t, *J* = 5.7 Hz, 0.4 H), 8.22 (t, *J* = 5.6 Hz, 0.6 H), 8.06 (d, *J* = 1.4 Hz, 0.6 H), 7.72–7.71 (m, 0.5 H), 7.68 (t, *J* = 5.5 Hz, 0.6 H), 7.61 (dd, *J* = 8.3 Hz, *J* = 1.5 Hz, 0.6 H), 7.55–7.49 (m, 4.8 H), 7.20–7.16 (m, 2.6 H), 7.10 (td, *J* = 7.7 Hz, *J* = 0.8 Hz, 2 H), 6.97 (t, *J* = 7.6 Hz, 2 H), 3.78–3.64 (m, 2 H), 3.62–3.48 (m, 10 H), 3.26–3.20 (m, 2 H), 2.90–2.85 (m, 6 H), 6.97 (t, *J* = 5.9 Hz, 2 H), 1.59–1.54 (m, 4 H), 1.50–1.46 (m, 27 H), 1.37–1.30 (m, 4 H), 1.13–1.10 (m, 12 H). ^13^C NMR (75 MHz, DMSO-*d*_6_, mixture of two isomers): *δ* [ppm] = 166.5, 166.2, 155.0, 153.7, 150.24, 150.21, 150.0, 149.9, 145.2, 142.8, 142.6, 132.4, 130.3, 130.2, 128.3, 126.7, 123.9, 123.23, 123.19, 119.9, 119.0, 115.7, 113.7, 85.8, 85.7, 85.3, 62.9, 58.2, 58.0, 53.6, 42.5, 42.3, 41.1, 30.7, 30.64, 30.61, 29.2, 27.4, 27.3, 26.2, 25.2, 24.4, 24.3, 22.6, 19.9, 19.8. ^31^P NMR (121 MHz, DMSO-*d*_6_, mixture of two isomers): *δ* [ppm] = 146.36, 147.35. IR: *ṽ* [cm^−1^] = 3383 (w), 2970 (w), 2931 (w), 2866 (w), 1720 (s), 1628 (m), 1601 (m), 1574 (s), 1537 (w), 1500 (w), 1456 (m), 1433 (w), 1394 (w), 1352 (m), 1335 (s), 1296 (w), 1281 (w), 1261 (m), 1215 (m), 1136 (s), 1080 (w), 1053 (w), 976 (w), 924 (w), 876 (w), 833 (w), 768 (m), 758 (m), 741 (m), 717 (m), 652 (m). MS (ESI+): *m*/*z* (%) = 1138.59 (100) [M + H]^+^, calcd for C_58_H_85_N_13_O_9_P: 1138.63.

*Di-tert-butyl-2,2’-((((2-((1-(tert-butoxycarbonyl)-6-((6-(((diisopropylamino)(methoxy)-phosphino)oxy)hexyl)carbamoyl)-1H-benzo[d]imidazol-2-yl)amino)ethyl)azanediyl)bis-(ethane-2,1-diyl))bis-(azanediyl))bis(1H-benzo[d]imidazole-1-carboxylate)* (**12**). To a solution of the alcohols **9a/b** (1.0 g, 1.07 mmol, 1 eq) and NEt_3_ (0.74 mL, 5.33 mmol, 5 eq) in dry DCM (15 mL) *N,N*-diisopropylmethylphosphonamidic chloride (0.32 g, 1.61 mmol, 1.5 eq) was added at 0 °C. After 15 min. at 0 °C and 30 min. at room temperature saturated NaHCO_3_ solution was added. The phases were separated and the aqueous layer was extracted with DCM. The extracts were dried over MgSO_4_, the solvent was removed under reduced pressure at 30 °C and the residue was purified via column chromatography (SiO_2_, EtOAc/MeOH 25:1 + 2% NEt_3_). Yield: 1.11 g (1.01 mmol, 94%). R_f_ = 0.31, 0.38 (EtOAc/MeOH 25:1 + 2% NEt_3_). ^1^H NMR (300 MHz, DMSO-*d*_6_, mixture of two isomers): ***δ*** [ppm] = 8.29 (t, *J* = 5.5 Hz, 0.4 H), 8.22 (t, *J* = 5.7 Hz, 0.6 H), 8.06 (d, *J* = 1.8 Hz, 0.6 H), 7.72 (t, *J* = 1.1 Hz, 0.4 H), 7.68 (t, *J* = 5.2 Hz, 0.6 H), 7.62 (dd, *J* = 8.4 Hz, *J* = 1.8 Hz, 1 H), 7.55–7.48 (m, 4.8 H), 7.20–7.16 (m, 2.6 H), 7.10 (td, *J* = 7.6 Hz, *J* = 1.2 Hz, 2 H), 6.97 (td, *J* = 7.6 Hz, *J* = 1.2 Hz, 2 H), 3.60–3.49 (m, 10 H), 3.27 (d, *J* = 13.0 Hz, 3 H), 3.27–3.20 (m, 2 H), 2.87 (t, *J* = 5.1 Hz, 6 H), 1.58–1.51 (m, 4 H), 1.50–1.46 (m, 27 H), 1.37–1.30 (m, 4 H), 1.10 (d, *J* = 6.8 Hz, 12 H). ^13^C NMR (75 MHz, DMSO-*d*_6_, mixture of two isomers): *δ* [ppm] = 166.5, 166.2, 154.9, 153.7, 150.2, 149.9, 145.2, 142.8, 142.6, 132.4, 130.7, 130.3, 130.2, 126.7, 123.9, 119.8, 115.7, 114.5, 114.3, 113.7, 85.7, 85.6, 85.3, 62.9, 62.6, 53.54, 53.47, 50.0, 49.8, 42.3, 42.1, 41.07, 41.04, 30.81, 30.78, 30.72, 29.3, 29.1, 27.4, 27.3, 26.2, 25.23, 25.20, 24.47, 24.41, 24.38, 24.31. ^31^P NMR (121 MHz, DMSO-*d*_6_): *δ* [ppm] = 146.98, 146.97. IR: *ṽ* [cm^−1^] = 3383 (w), 2970 (w), 2931 (w), 2866 (w), 1720 (s), 1628 (m), 1601 (m), 1574 (s), 1537 (w), 1500 (w), 1456 (m), 1433 (w), 1394 (w), 1352 (m), 1335 (s), 1296 (w), 1281 (w), 1261 (m), 1215 (m), 1136 (s), 1080 (w), 1053 (w), 976 (w), 924 (w), 876 (w), 833 (w), 768 (m), 758 (m), 741 (m), 717 (m), 652 (m). MS (ESI+): *m*/*z* (%) = 1099.60 (100) [M + H]^+^, calcd for C_56_H_84_N_12_O_9_P: 1099.62.

### 4.2. Synthesis, Purification, Quantification and Analysis of Conjugates 

All of the DNA-LNA mixmers required to prepare conjugates **13**–**20** were purchased from Eurogentec, Seraing, Belgium and delivered when still bound to the solid support. Reversed Phase (RP) HPLC was executed on a Jasco LC-900 HPLC system (Jasco Germany GmbH, Pfungstadt, Germany) equipped with a Jasco UV-975 detector (detection at 254 nm) and a semi preparative column Phenomenex Jupiter 4 μm Proteo 90 Å (250 × 10 mm). All of the conjugates were lyophilized. Water was treated with DEPC and autoclaved.

#### 4.2.1. Manual Phosphoramidite Coupling 

Phosphoramidite (40 mg per 1 µmol of oligonucleotide) and oligo on CPG were placed in a syringe and dried in vacuo. The syringe was repeatedly flushed with argon and 1.5 mL of a 0.5 M solution of 5-ethylthio-1*H*-tetrazole (ETT) in dry MeCN was added. After shaking at room temperature for 15 min., the solution was discarded and the CPG was washed several times with MeCN. 200 µL oxidizer (2% I_2_ in pyridine/water) were added and then shaken for 45 s. Excess oxidizer was removed by repeatedly washing with MeCN.

#### 4.2.2. Isolation and Purification 

For cleavage from solid support, the CPG was treated with NH_4_OH/EtOH (3:1) twice for 30 min. at room temperature and the solutions were incubated at 50 °C over night. RP-HPLC: A: 1 M TEAA buffer pH 7.0, B: acetonitrile, C: DEPC-H_2_O. Gradient: constant 10% A, 3% B from 0–1 min, 3–33% from 1–20 min.; flow 4 mL/min.; and, column temperature 50 °C.

#### 4.2.3. Quantification

Oligonucleotide concentrations were determined via UV spectrometry on a nanodrop2000 (Thermo Scientific, Waltham, MA, USA) using Lambert–Beer´s law. Extinction coefficients were calculated by a nearest neighbor model according to literature [42]. For simplification, influences of the tris(2-aminobenzimidazole) part were neglected.

#### 4.2.4. Mass spectrometry

Oligonucleotides were analyzed via ESI mass spectrometry while using a LCMS instrument with microTOF-Q II analyser (Bruker Daltonics, Bremen, Germany). An Agilent 1200 Series HPLC using methanol/0.005 M TEAA buffer (gradient 0–60%) was applied as LC system.

#### 4.2.5. Cleavage Studies

For saturation experiments [36] a solution containing 150 nM Cy5 labeled RNA, 0–3 µM conjugate and 50 mM Tris buffer (pH 8.0) was mixed in an Eppendorf tube (DNA LoBind Tubes) to reach a final volume of 10 µL. This tube was kept at 37 °C for 20 h. The cleavage reactions were stopped by addition of 15 µL loading buffer (8 M urea, 20 mM EDTA, 0.2% crocein orange) and samples were kept at −196 °C. For analysis, 1 µL of this solution was added to an ALFexpress sequencer (see Supplementary Materials).

For kinetic measurements, a solution containing 150 nM Cy5 labeled RNA, 3 µM conjugate, and 50 mM Tris buffer (pH 8.0) was mixed in an Eppendorf tube (DNA LoBind Tubes) to reach a final volume of 200 µL. This tube was kept at 37 °C and samples of 10 µL were drawn after indicated time intervals. The cleavage reactions were stopped by addition of 15 µL loading buffer (8 M urea, 20 mM EDTA, 0.2% crocein orange) and samples were kept at −196 °C. For analysis, 1 µL of this solution was added to an ALFexpress sequencer.

To prepare an RNA hydrolysis ladder, a solution of 375 nM RNA and 0.25 mM Na_2_CO_3_ was mixed in an Eppendorf tube (DNA LoBind Tubes) to reach a final volume of 8 µL. This tube was kept at 90 °C for 12 min. After cooling in an ice bath, the reaction was stopped by addition of 2 µL 1 M AcOH and 15 µL loading buffer (8 M urea, 20 mM EDTA, 0.2% crocein orange). The samples were stored at −20 °C until usage.

## Figures and Tables

**Table 1:** Kinetics and site-selectivity of RNA cleavage by conjugates **3**–**20**.

Conjugate	*t* ^1/2^	n − 2	n − 1	n	n + 1	n + 2	n + 3
**3** ^1,3^	14 ± 0.9 h	1.4%	2.5%	**89.0**%	7.1%		
**4** ^1,3^	3.5 ± 0.4 h	1.6%	5.8%	**83.9**%	8.6%		
**13** ^1^	13.3 ± 0.8 h		1.5%	**65.2**%	30.2%	3.1%	
**14** ^1^	6.4 ± 0.2 h		1.2%	**75.3**%	22.1%	1.4%	
**15** ^1^	6.5 ± 0.5 h		1.5%	**88.3**%	10.1%		
**16** ^1^	5.6 ± 0.1 h		1.2%	**74.9**%	22.1%	1.7%	
**5** ^2,3^	15.0 ± 1.7 h	29.0%	22.7%	**35.8**%	5.1%	3.6%	
**6** ^2,3^	10.0 ± 0.3 h	6.4%	35.0%	**45.9**%	6.9%	4.5%	1.3%
**17** ^2^	18.3 ± 1.2 h	5.2%	4.1%	**52.1**%	22.5%	9.6%	2.3%
**18** ^2^	14.2 ± 0.7 h		2.4%	**67.4**%	21.8%	6.1%	2.2%
**19** ^2^	13.0 ± 1.5 h		10.7%	**71.3**%	9.8%	6.4%	1.8%
**20** ^2^	9.8 ± 1.0 h		3.2%	**72.4**%	17.2%	3.4%	1.1%

Determined by peak integration of all cleavage products. **n** means cleavage between nucleotides 15 and 16, directly after the duplex region. ^1^ Reaction with RNA **1**. ^2^ Reaction with RNA **2**. ^3^ Taken from reference 39.

## Supplementary Materials

The following are available online, HPLC plots and mass spectra of conjugates **13**–**20**. Removal of Boc by ammonolysis. Description of the ALFexpress sequencer. NMR spectra.
