# Supplementary material for: A Trisbenzimidazole Phosphoramidite Building Block Enables High-Yielding Syntheses of RNA-Cleaving Oligonucleotide Conjugates
Source: Molecules. 2020 Apr 16;25(8):1842. doi: 10.3390/molecules25081842 (PMC7221912; doi:10.3390/molecules25081842)
Supplement: Supplementary file 1 [file molecules-25-01842-s001.pdf]

Supporting Information

A Trisbenzimidazole Phosphoramidite Building Block Enables High-yielding Syntheses of RNA-cleaving Oligonucleotide Conjugates

Felix Zellmann and Michael W. Göbel*

Institute of Organic Chemistry and Chemical Biology, Goethe University Frankfurt
Max-von-Laue-Str. 7, D-60438 Frankfurt am Main, Germany
Email: Michael W. Göbel – m.goebel@chemie.uni-frankfurt.de

* Corresponding author

Table of contents:

Comparison of conjugation methods……………………………………………………………..2
HPLC and mass spectra of conjugates……………………………………………………………2
Boc-deprotection of **9a**/**b**... 11
Analysis of RNA cleavage using the ALFexpress sequencer……………………………………13
NMR spectra………………………………………………………………………………………15

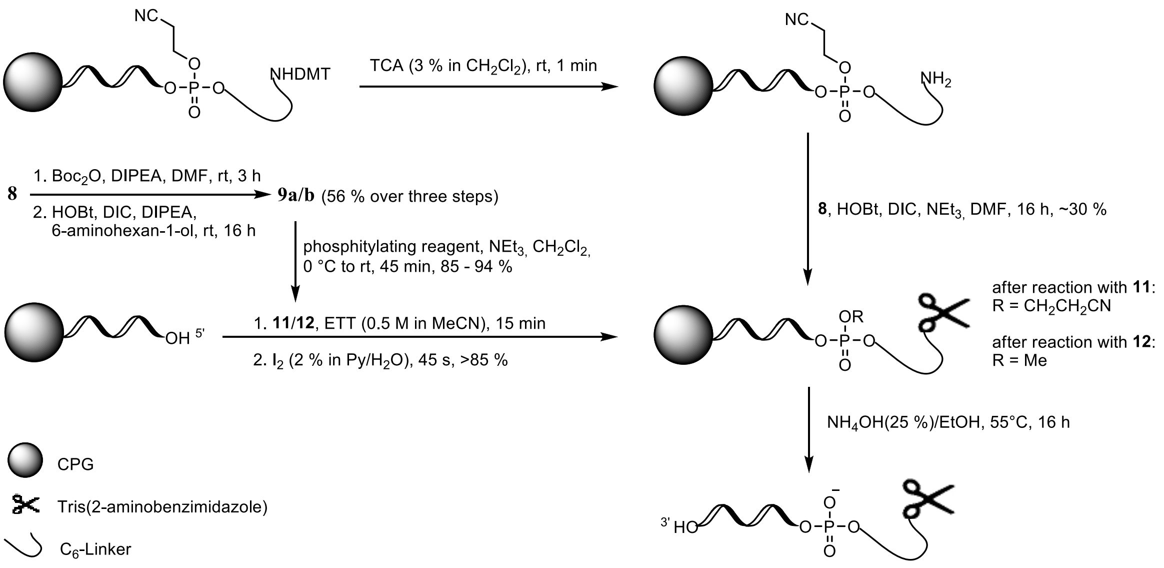

Figure S1: Comparison of our previous conjugation method (active ester coupling with an amino linker) and the new phosphoramidite approach resulting in largely improved yields.

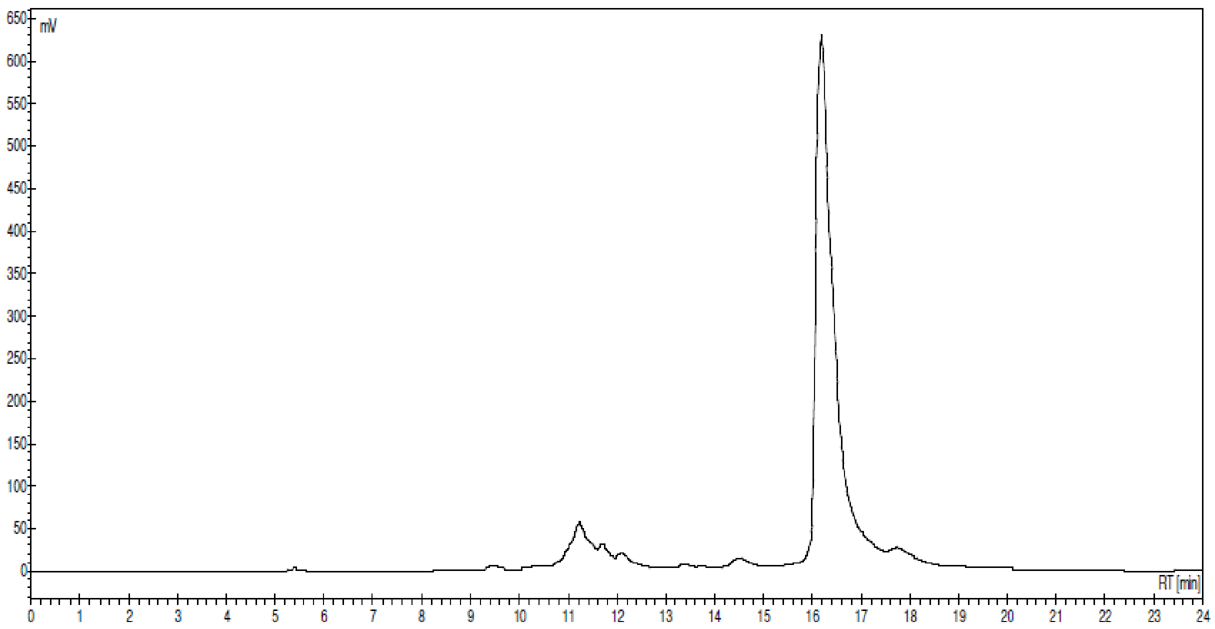

Figure S2: Typical chromatogram of a crude conjugate after coupling of phosphoramidite **12**. Determination of coupling yields by HPLC usually gave values > 85 %.

Conjugate **13** calculated exact mass: 5387.1; found 5387.2

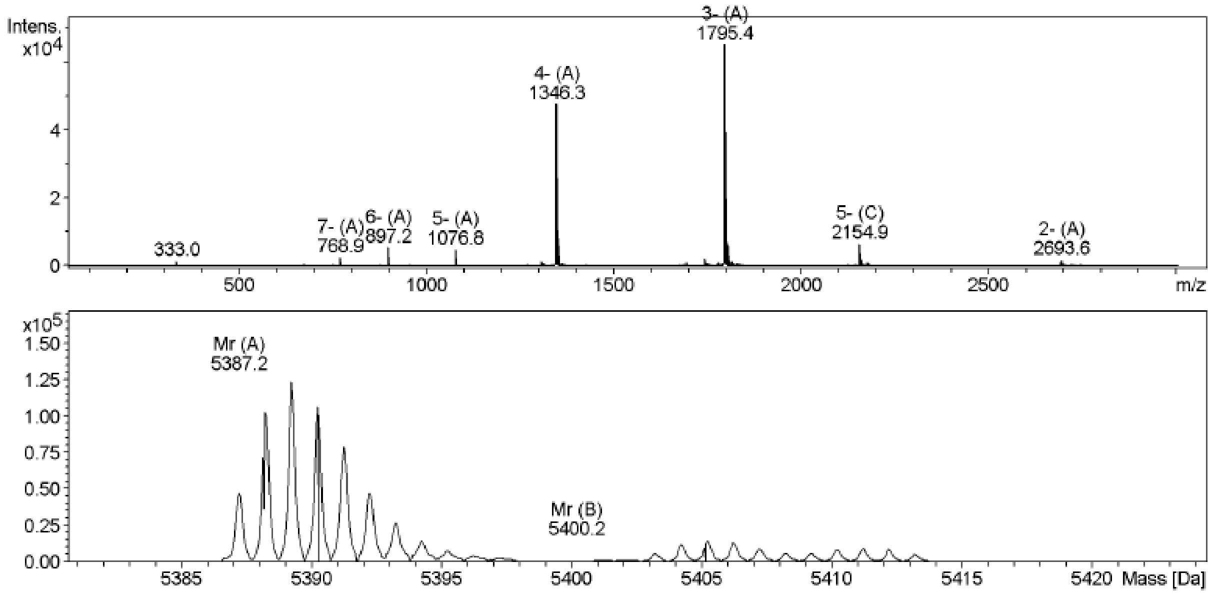

Figure S3: LC-MS analysis of conjugate **13**.

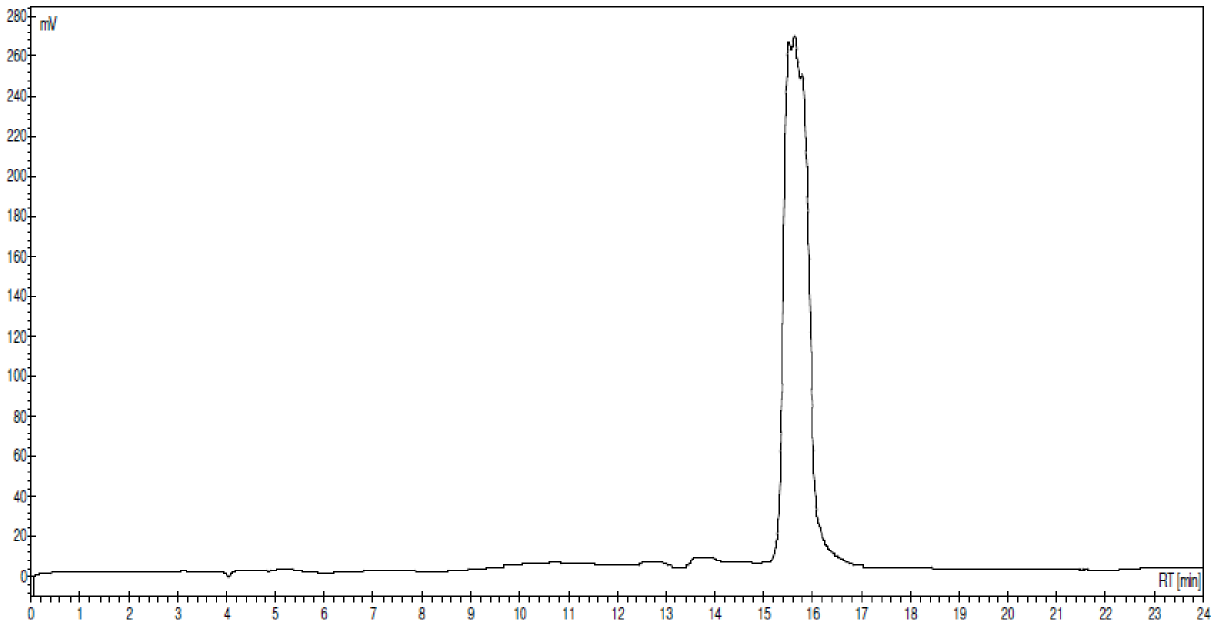

Figure S4: Chromatogram of conjugate **13** after purification.

Conjugate **14** calculated exact mass: 5415.1; found 5415.2

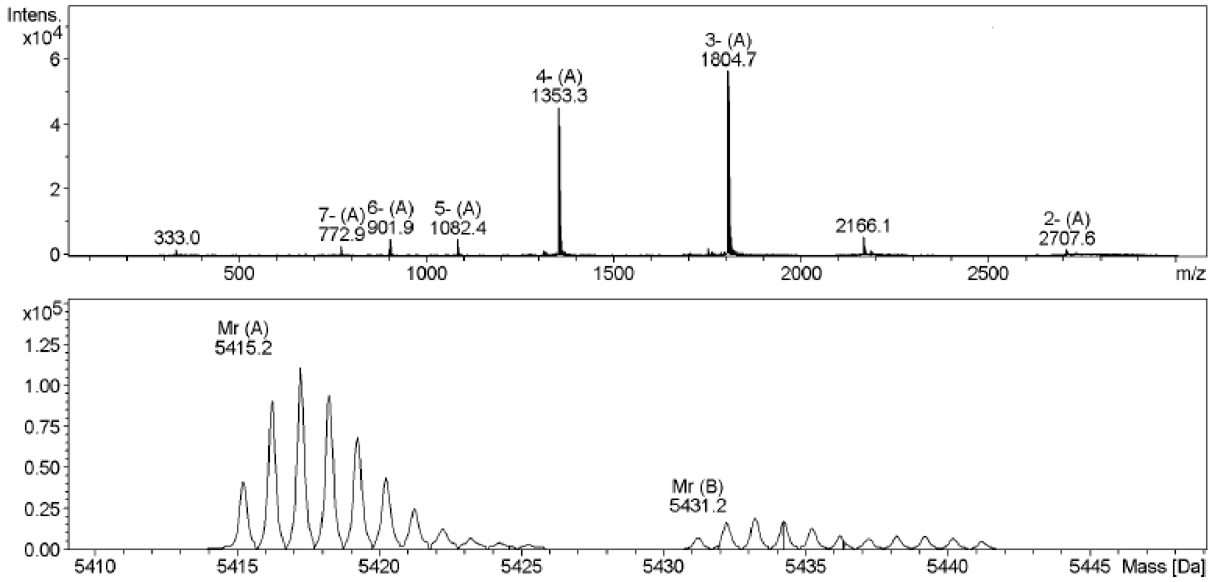

Figure S5: LC-MS analysis of conjugate **14**.

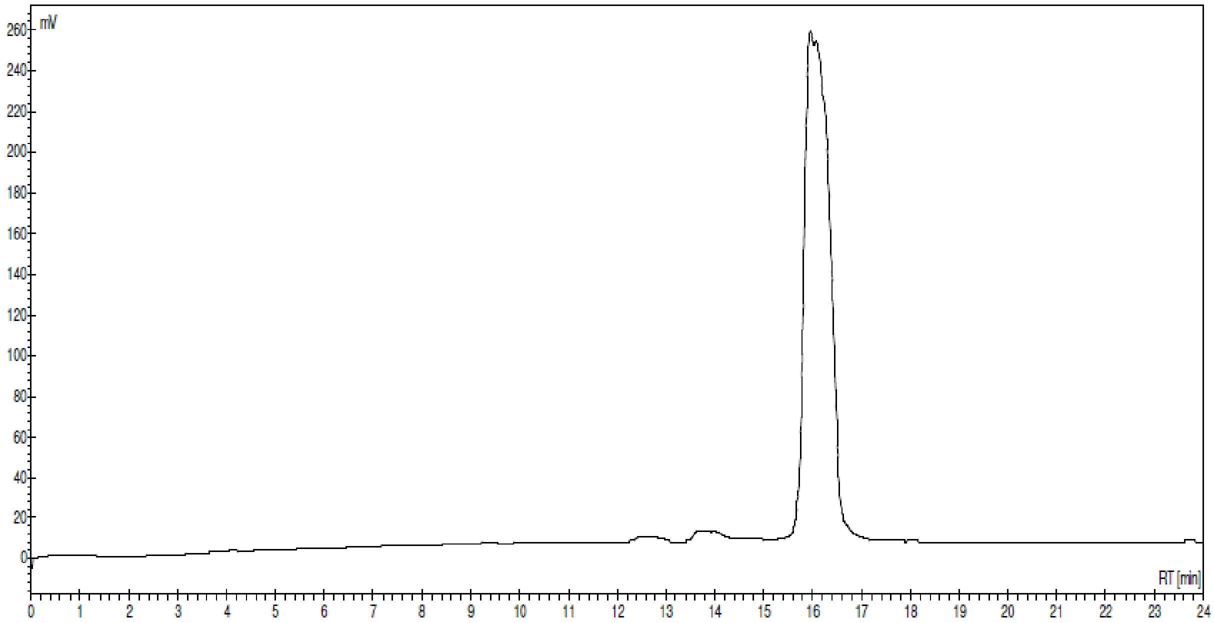

Figure S6: Chromatogram of conjugate **14** after purification.

Conjugate **15** calculated exact mass: 5415.1; found 5415.2

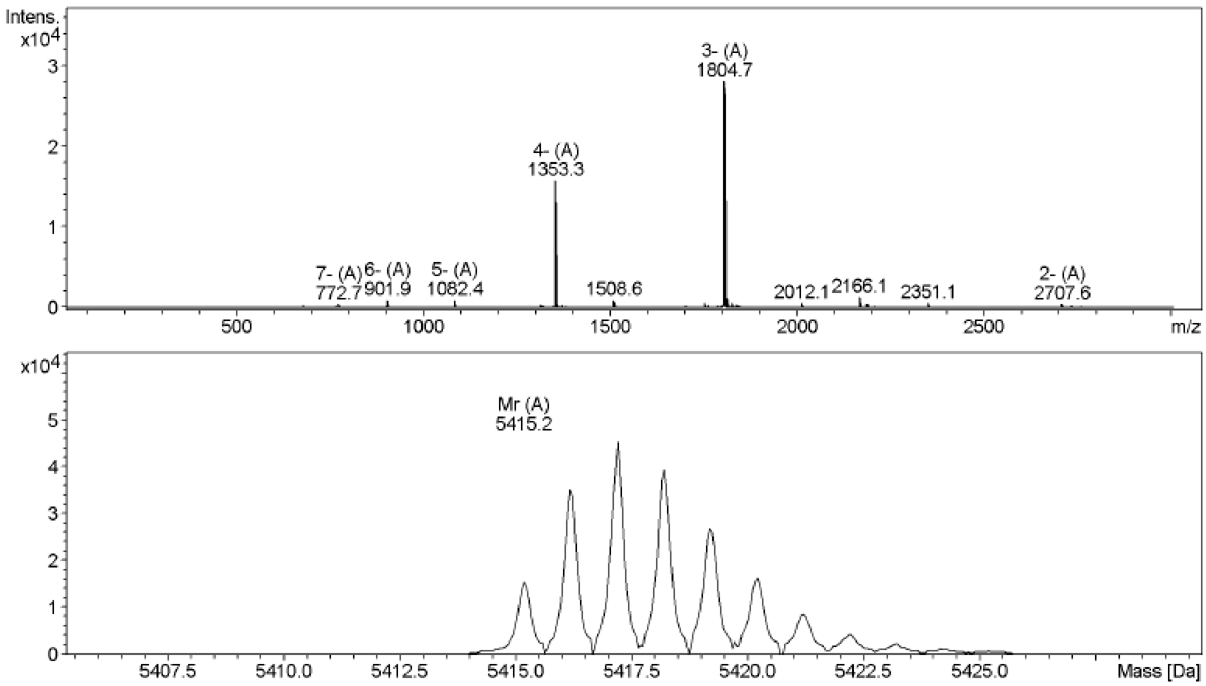

Figure S7: LC-MS analysis of conjugate **15**.

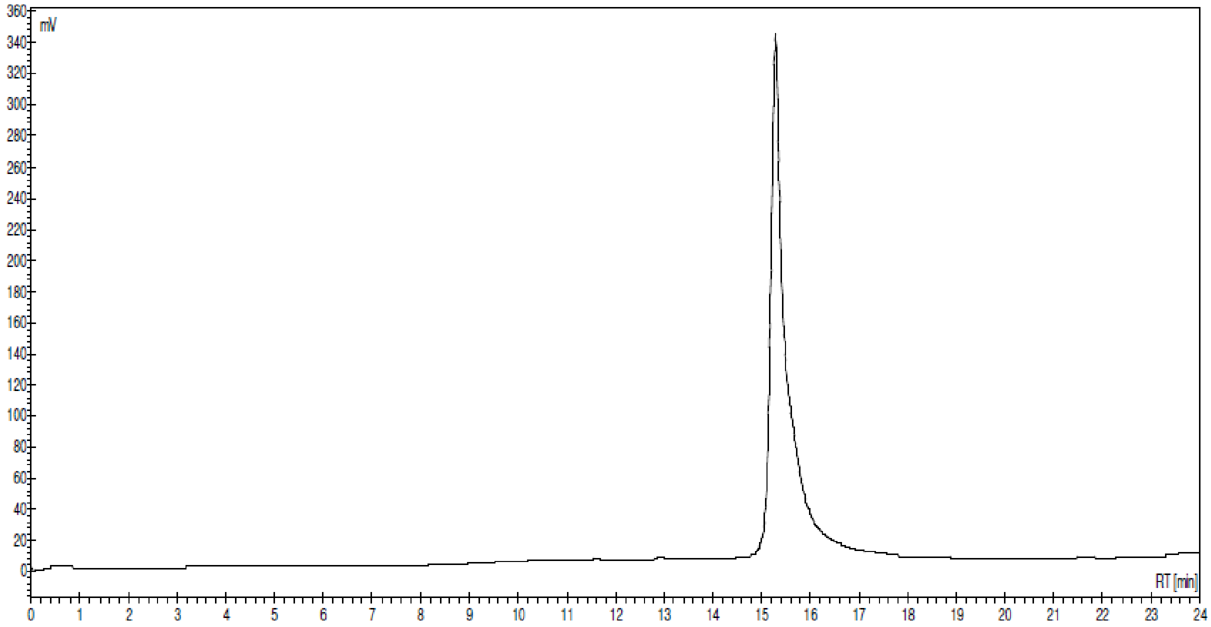

Figure S8: Chromatogram of conjugate **15** after purification.

Conjugate **16** calculated exact mass: 5443.1; found 5443.2

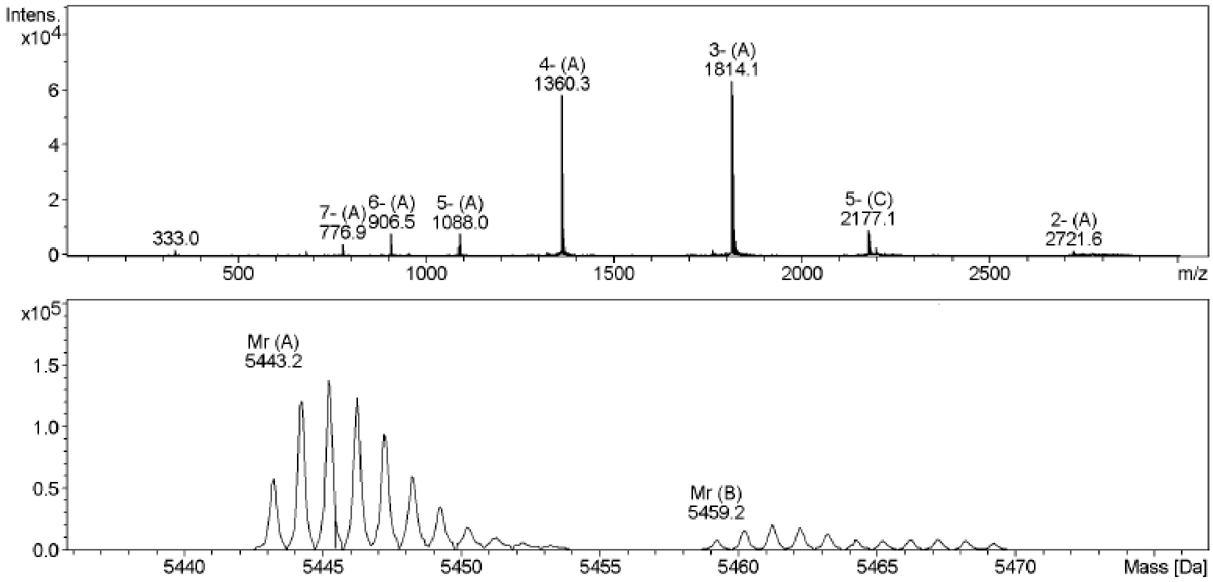

Figure S9: LC-MS analysis of conjugate **16**.

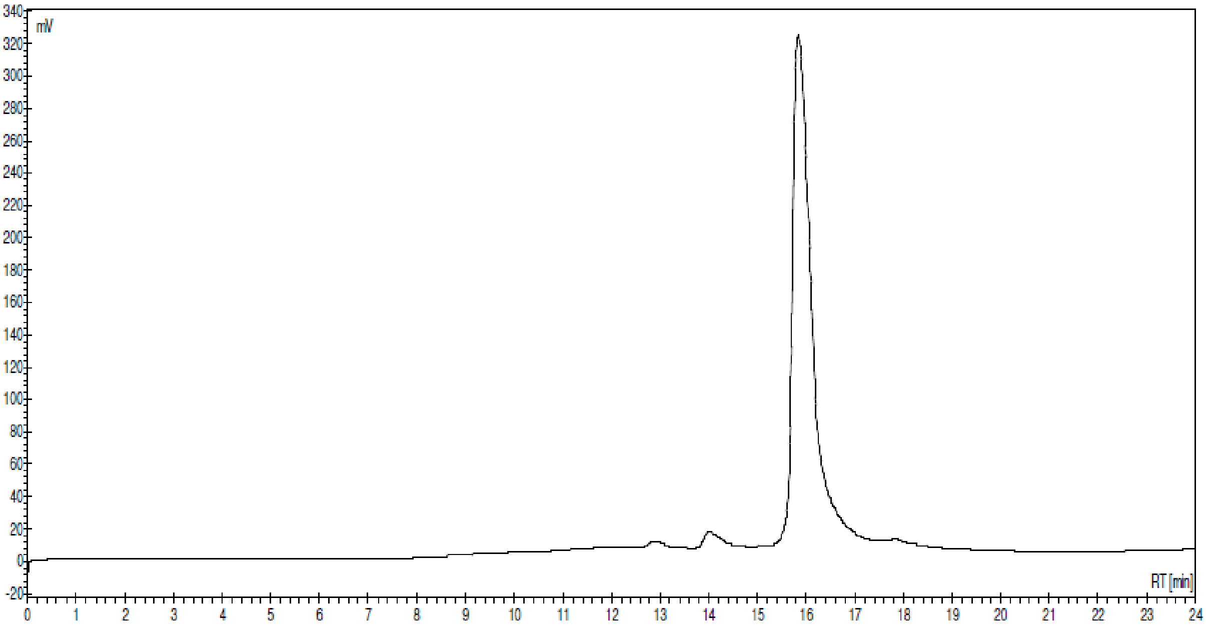

Figure S10: Chromatogram of conjugate **16** after purification.

Conjugate **17** calculated exact mass: 5305.1; found 5305.1

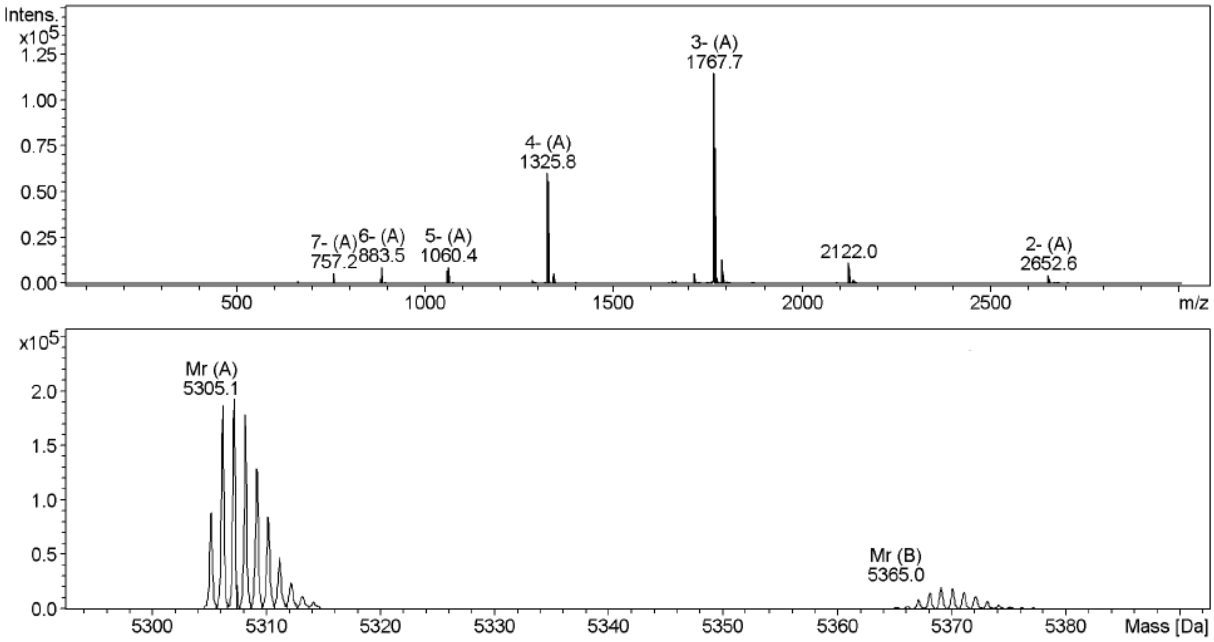

Figure S11: LC-MS analysis of conjugate **17**.

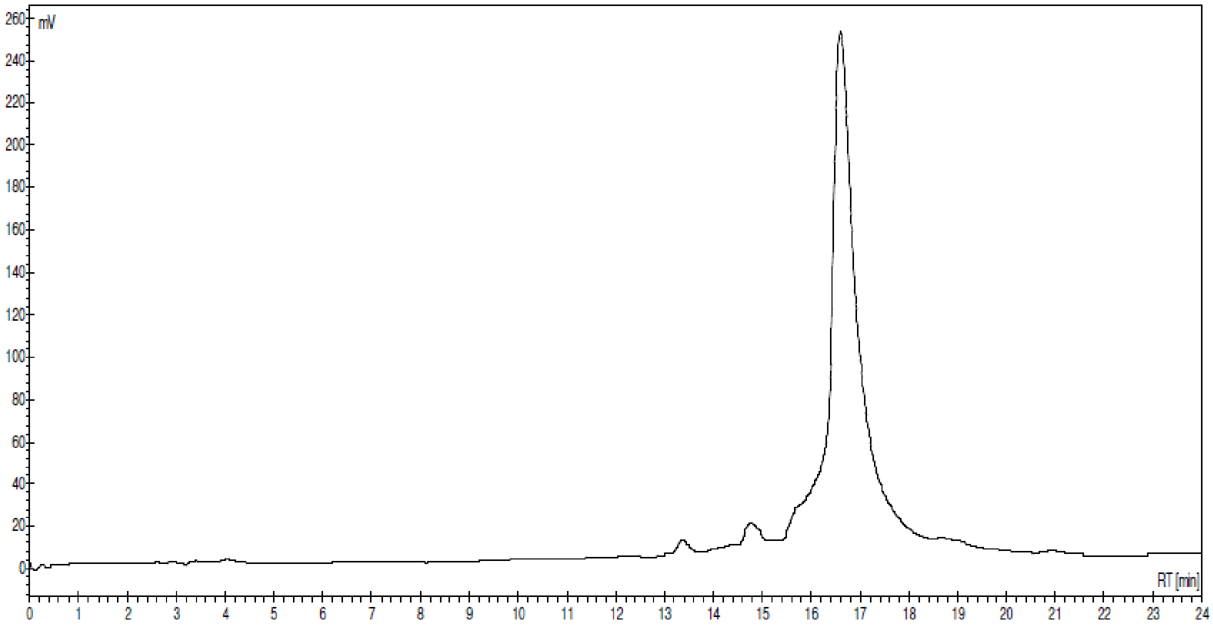

Figure S12: Chromatogram of conjugate **17** after purification.

Conjugate **18** calculated exact mass: 5333.1; found 5333.1

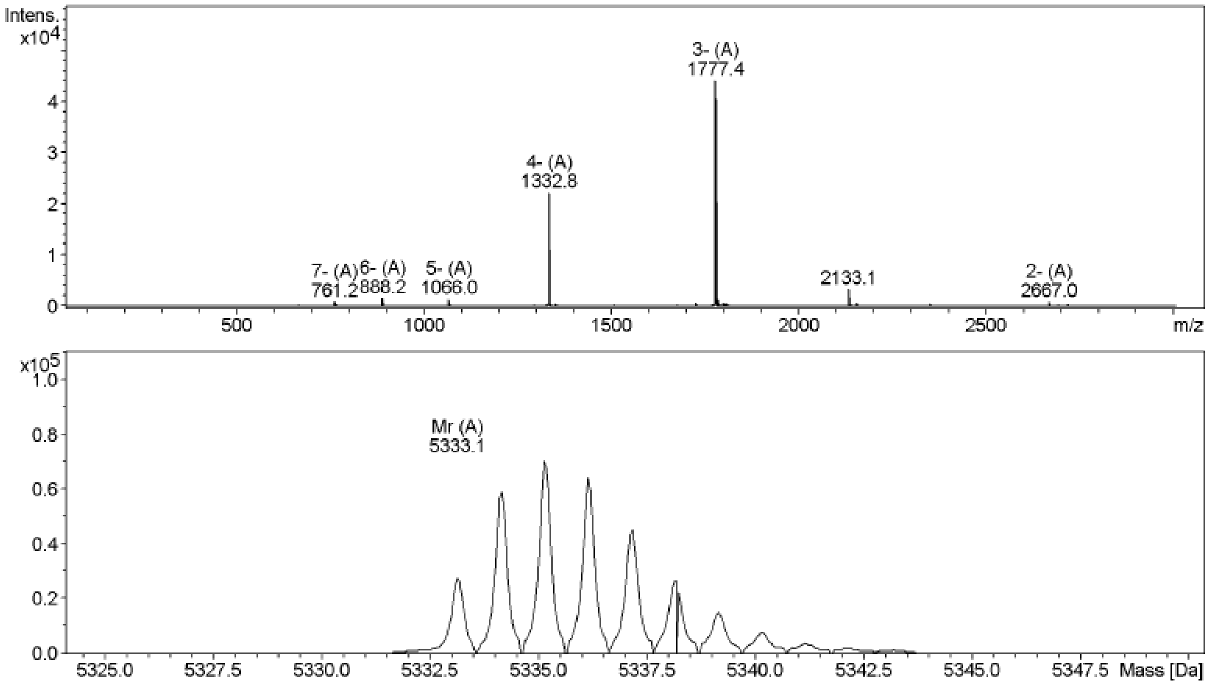

Figure S13: LC-MS analysis of conjugate **18**.

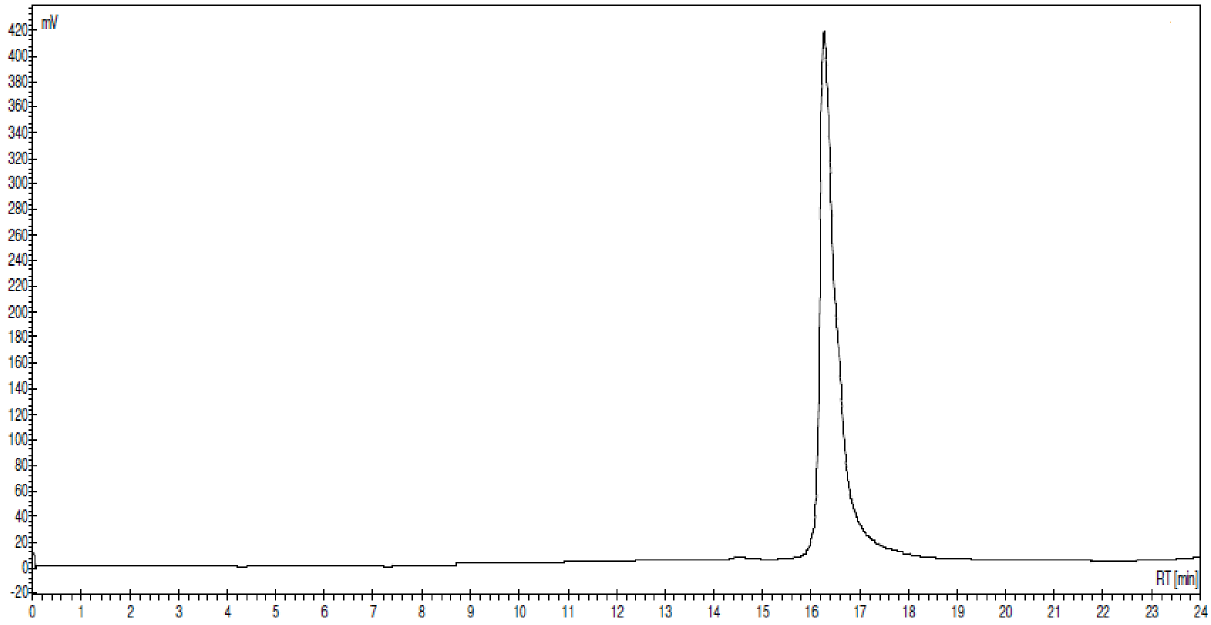

Figure S14: Chromatogram of conjugate **18** after purification.

Conjugate **19** calculated exact mass: 5333.1; found 5333.1

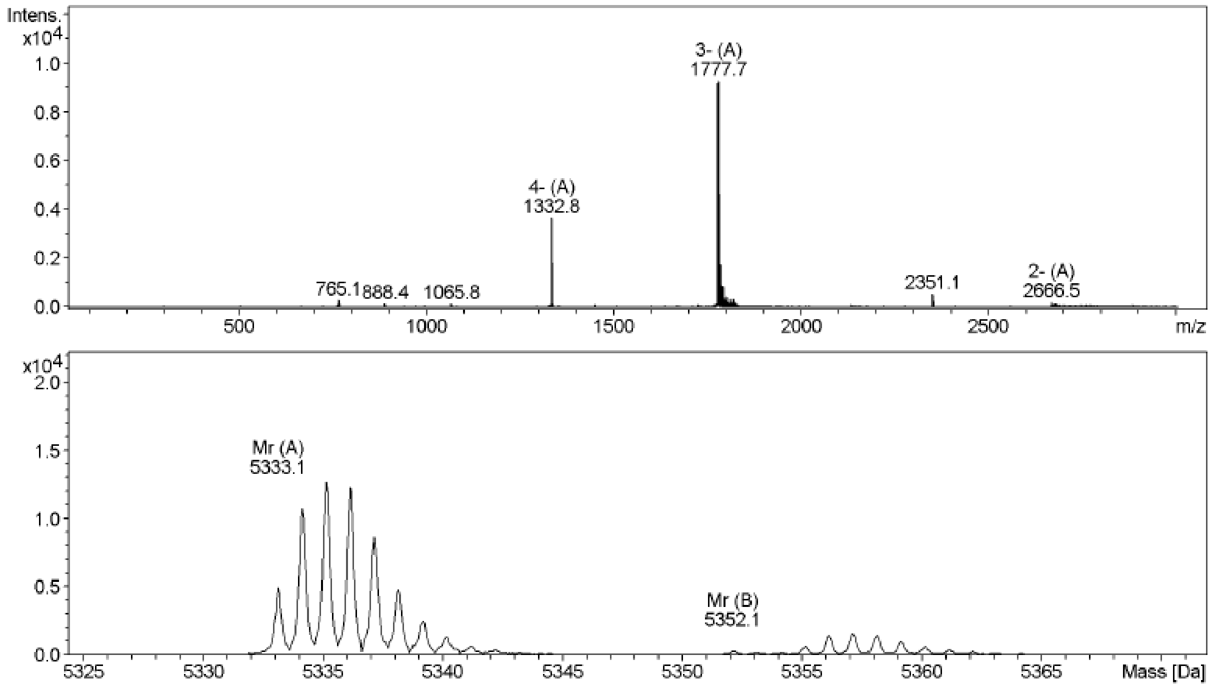

Figure S15: LC-MS analysis of conjugate **19**.

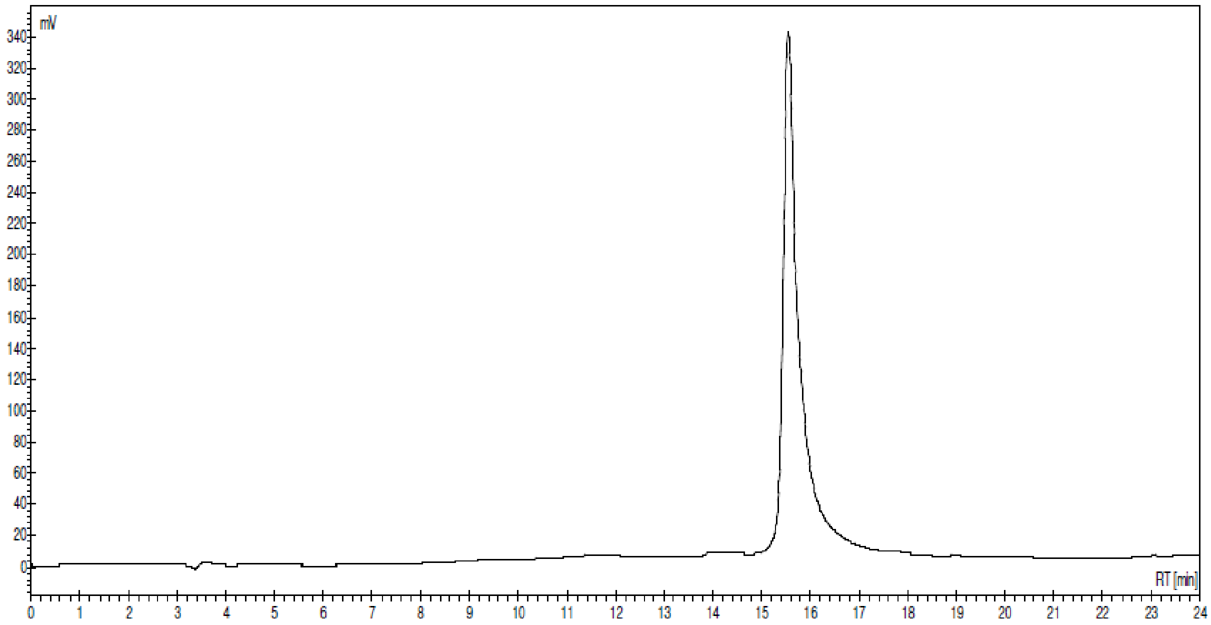

Figure S16: Chromatogram of conjugate **19** after purification.

Conjugate **20** calculated exact mass: 5361.1; found: 5361.1

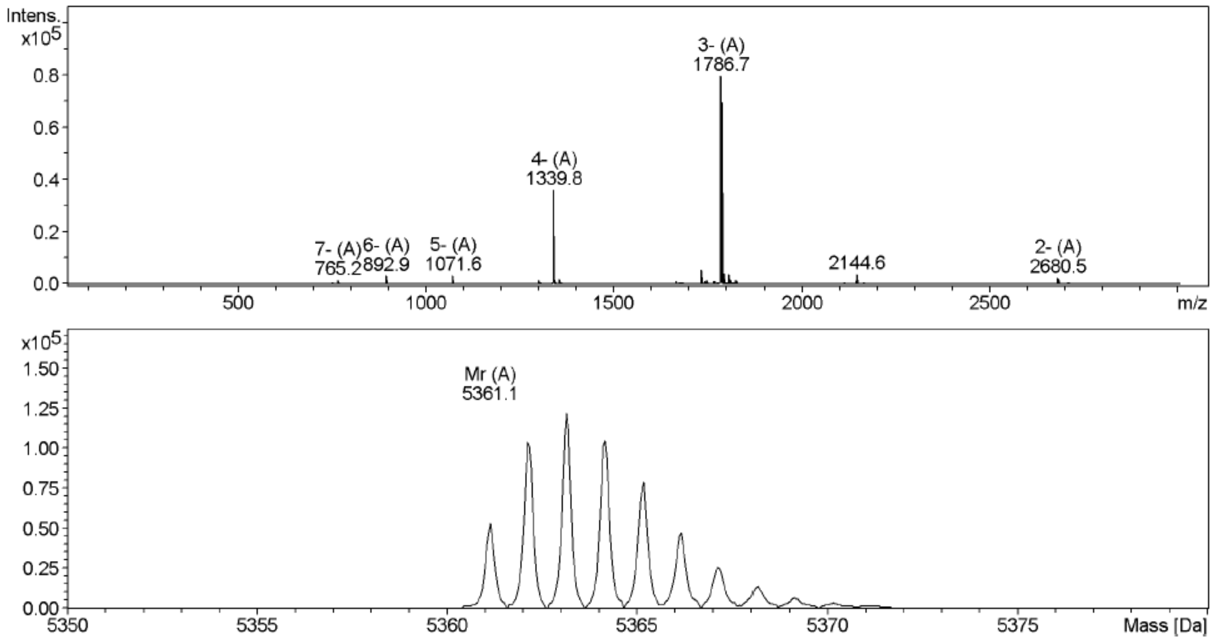

Figure S17: LC-MS analysis of conjugate **20**.

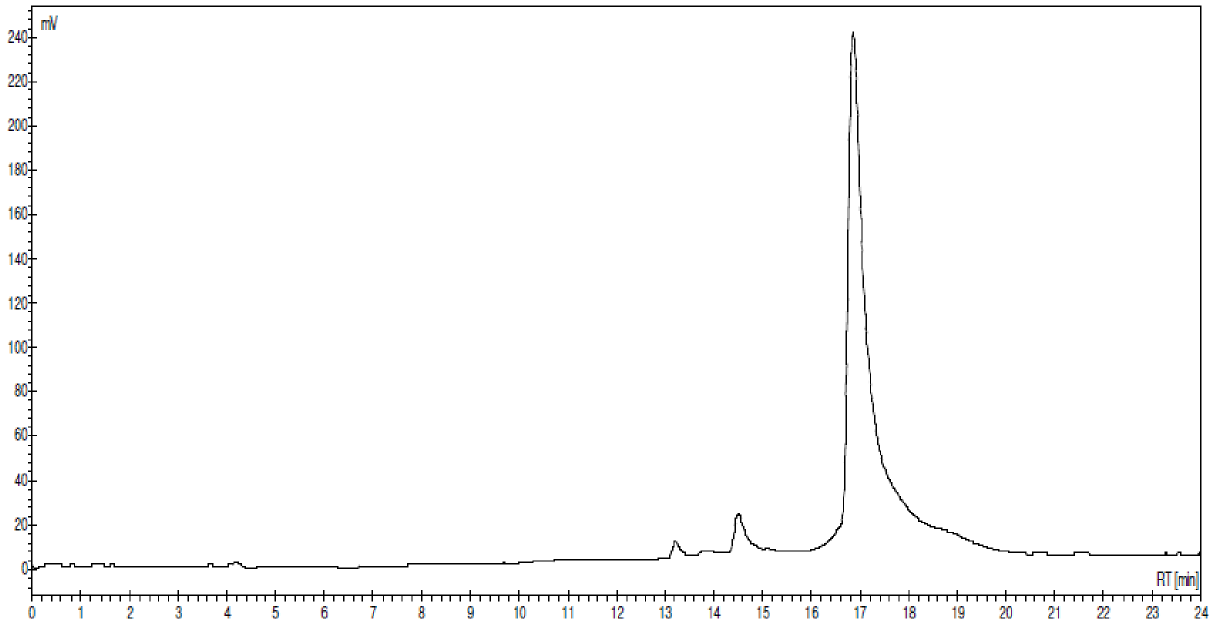

Figure S18: Chromatogram of conjugate **20** after purification.

Boc-deprotection of 9a/b

For Boc-deprotection studies of **9a/b** 10 mg of the compound mixture was taken up in 0.5 mL NH_4OH solution (25 %) and EtOH was added until no more precipitate could be observed. This solution was incubated at 37 °C or at 55 °C, respectively. After given times, samples were taken, brine was added and the solution was extracted with EtOAc. Phases were separated and the organic phase was analyzed by RP-HPLC.

Reversed Phase (RP) HPLC was executed on a Jasco LC-900 HPLC system equipped with a Jasco UV-975 detector (detection at 254 nm) and an analytical column Macherey Nagel NUCLEODUR C18 HTec 5 µm (150 x 4 mm).

RP-HPLC: A: 5 mM $NH_4H_2PO_4$ in water, B: acetonitrile. Gradient: 20 % B from 0-5 min, 20-90 % B from 5-10 min, 90 % B from 10-30 min; flow 0.7 mL/min.

Table S1: Conversion rate of **9a/b** by reaction with NH_4OH solution (25 %). Yields of **10** were determined by HPLC.

T \ t	**2 h**	**4 h**	**6 h**	**8 h**	**16 h**
**37 °C**	-	-	-	-	50 %
**55 °C**	29 %	59 %	77 %	77 %	100 %

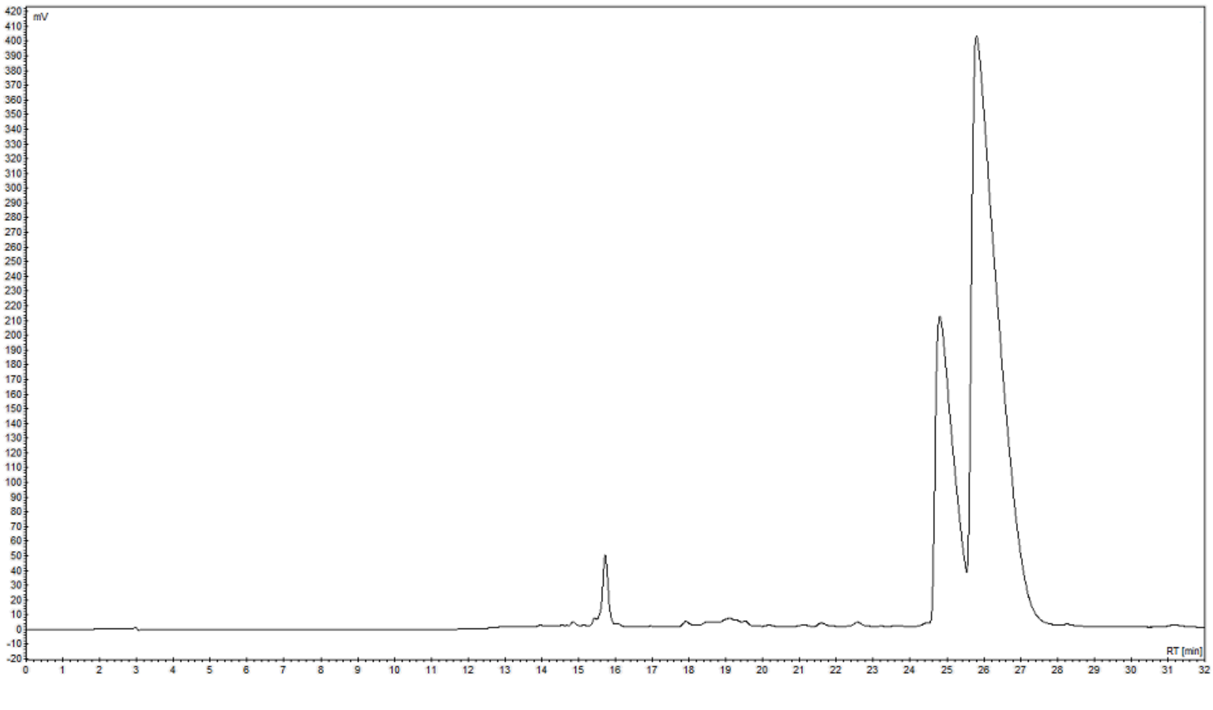

Figure S19: Chromatogram of **9a/b** before deprotection.

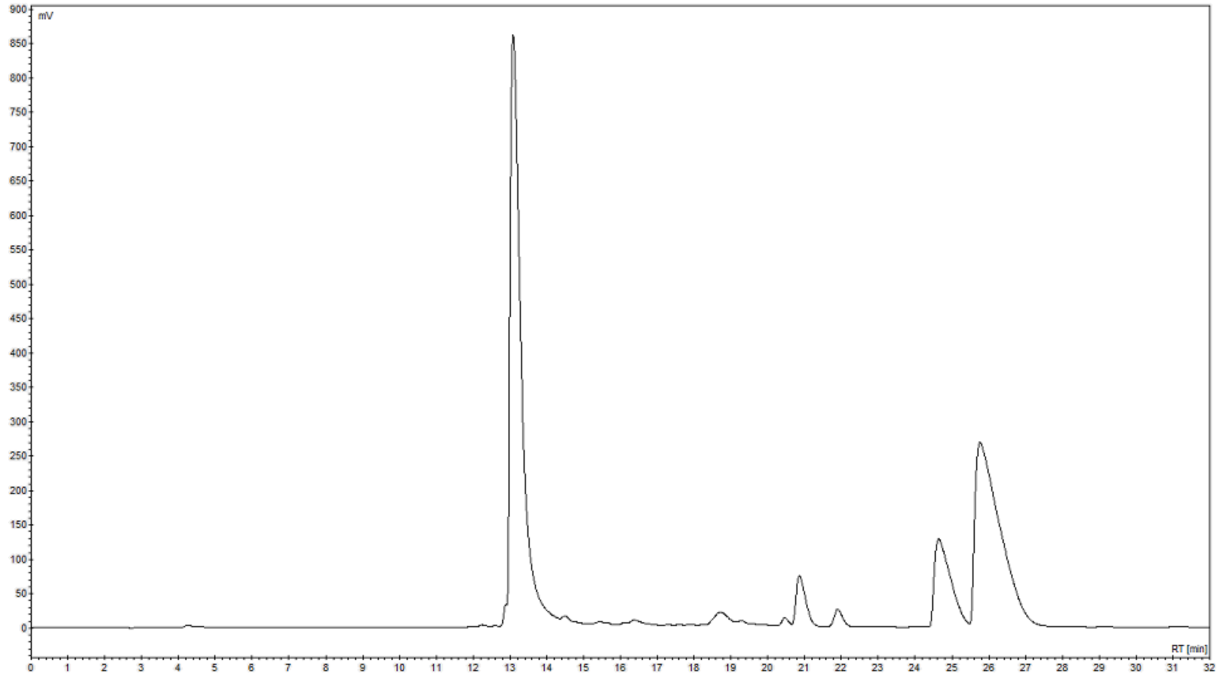

Figure S20: Chromatogram of **9a**/**b** after 16 h at 37 °C. Deprotection is incomplete.

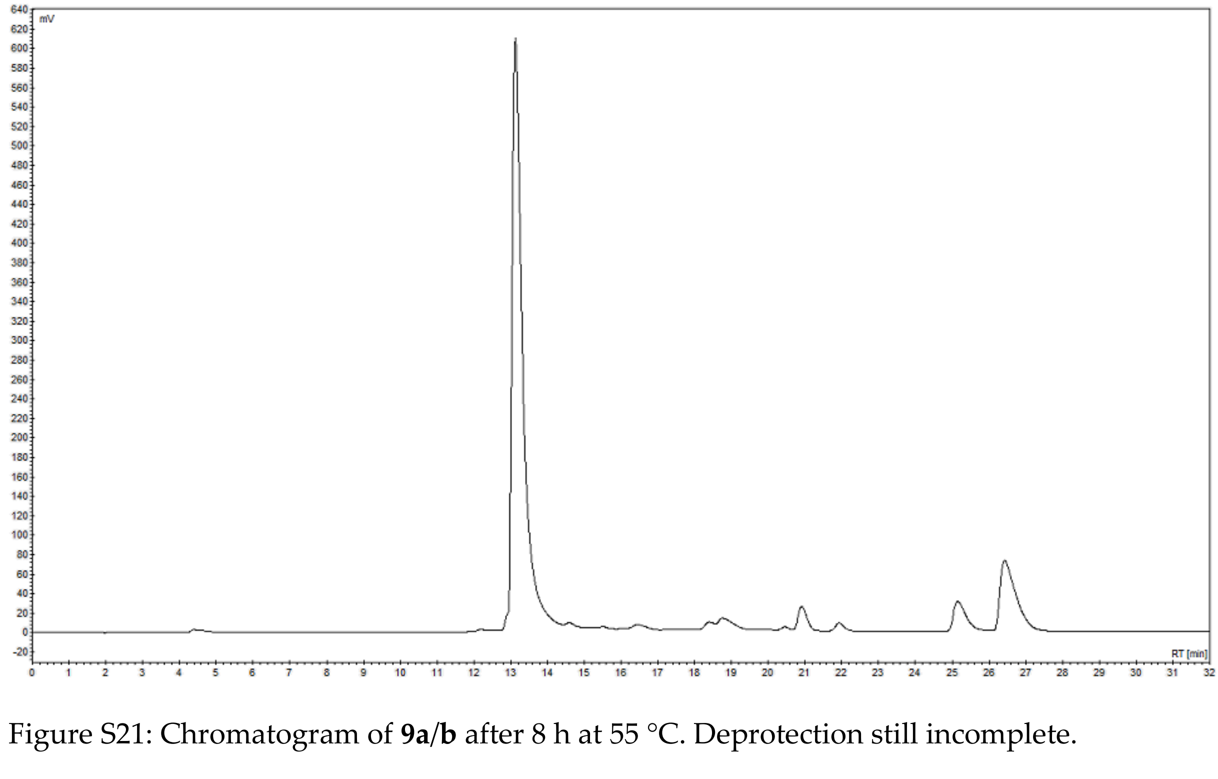

Figure S21: Chromatogram of **9a**/**b** after 8 h at 55 °C. Deprotection still incomplete.

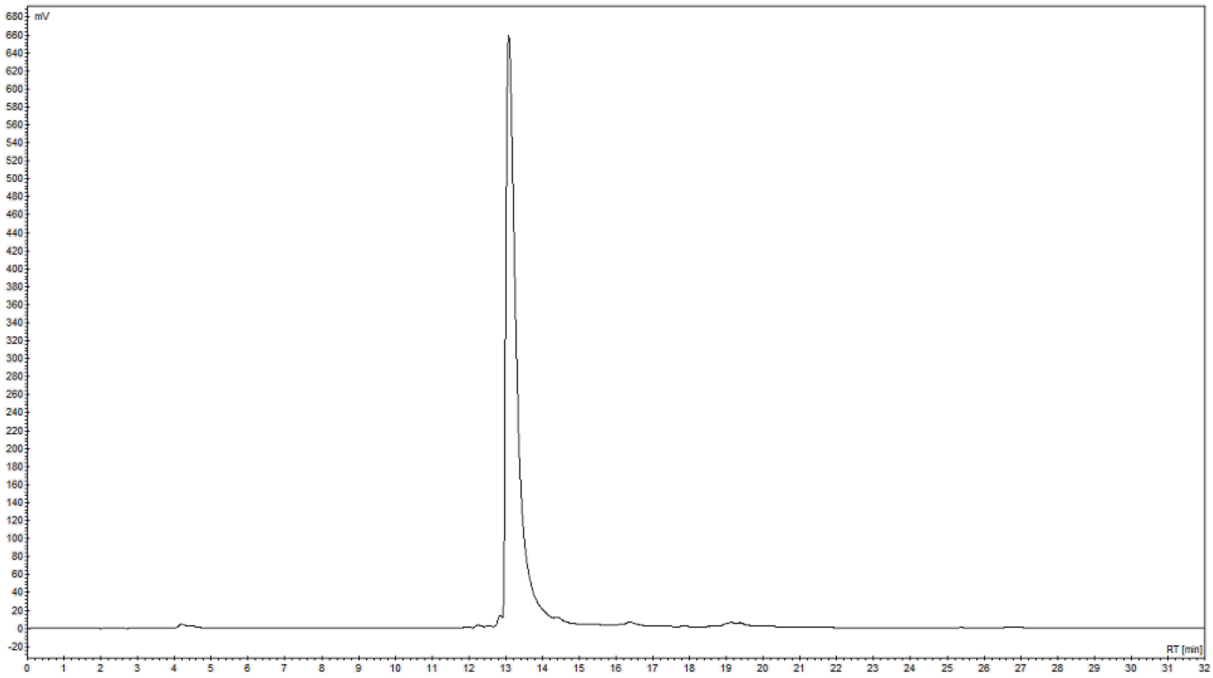

Figure S22: Chromatogram of **9a**/**b** after 16 h at 55 °C. Removal of Boc completed.

Analysis of RNA cleavage using the ALFexpress sequencer

The ALFexpress sequencer performs denaturing polyacrylamide gel electrophoresis under well controlled conditions. Figure S23 shows the instrument in the open state with the gel cassette removed. Cy5-labeled samples are loaded into 40 lanes on the top of the gel. Upon electrophoresis, dye labeled oligonucleotides and cleavage fragments move downwards in the gel with different speeds until they cross a horizontal laser beam at the bottom of the sequencer. Fluorescent emission of Cy5 is then registered independently for each lane by 40 avalanche photodiodes (shiny oval spots down in Figure S23). As long as the dye-labeled oligonucleotides do not contain quenching chromophores, the signal is proportional to the absolute concentration of Cy5 moving through the laser beam. Due to attenuation of the beam by the gel matrix, identical samples may generate stronger signals in lane 1 compared

to lane 40. Within a single lane, however, integrals of the fluorescent signals represent the relative concentration of each Cy5-labeled species present in that lane. Thus, although the method is denaturing polyacrylamide gel electrophoresis, the plotted data look like HPL chromatograms and can be interpreted in the same way. The cleavage patterns shown in Figures 2a and 4a were obtained in this way.

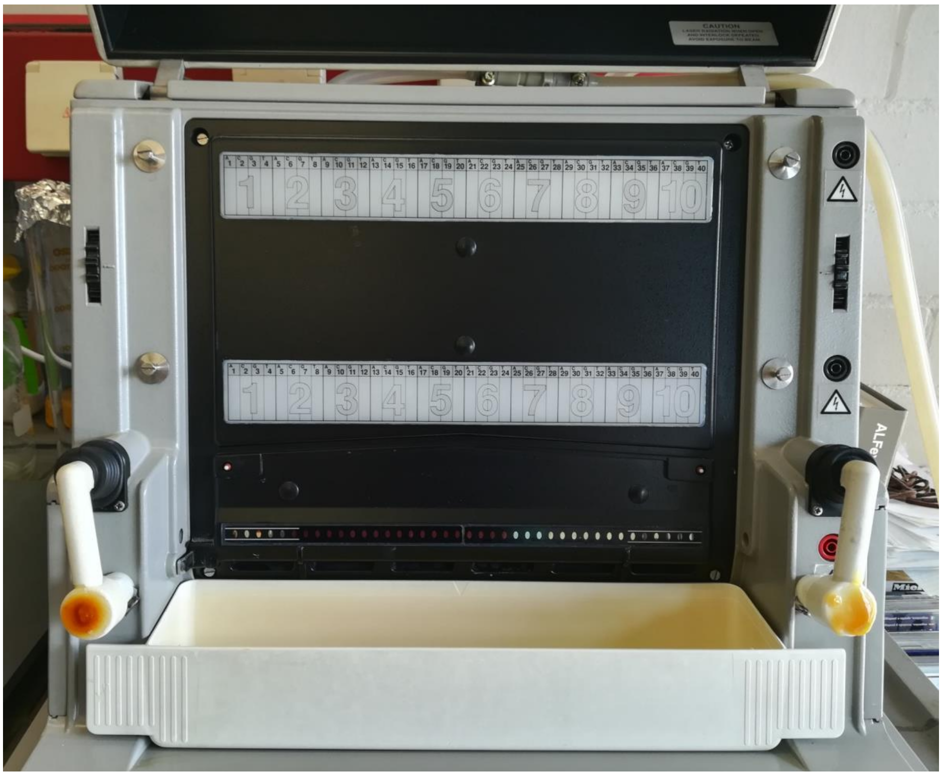

Figure S23: Front view of ALFexpress sequencer (open, gel chamber removed) used to analyze the cleavage of dye-labeled RNA.

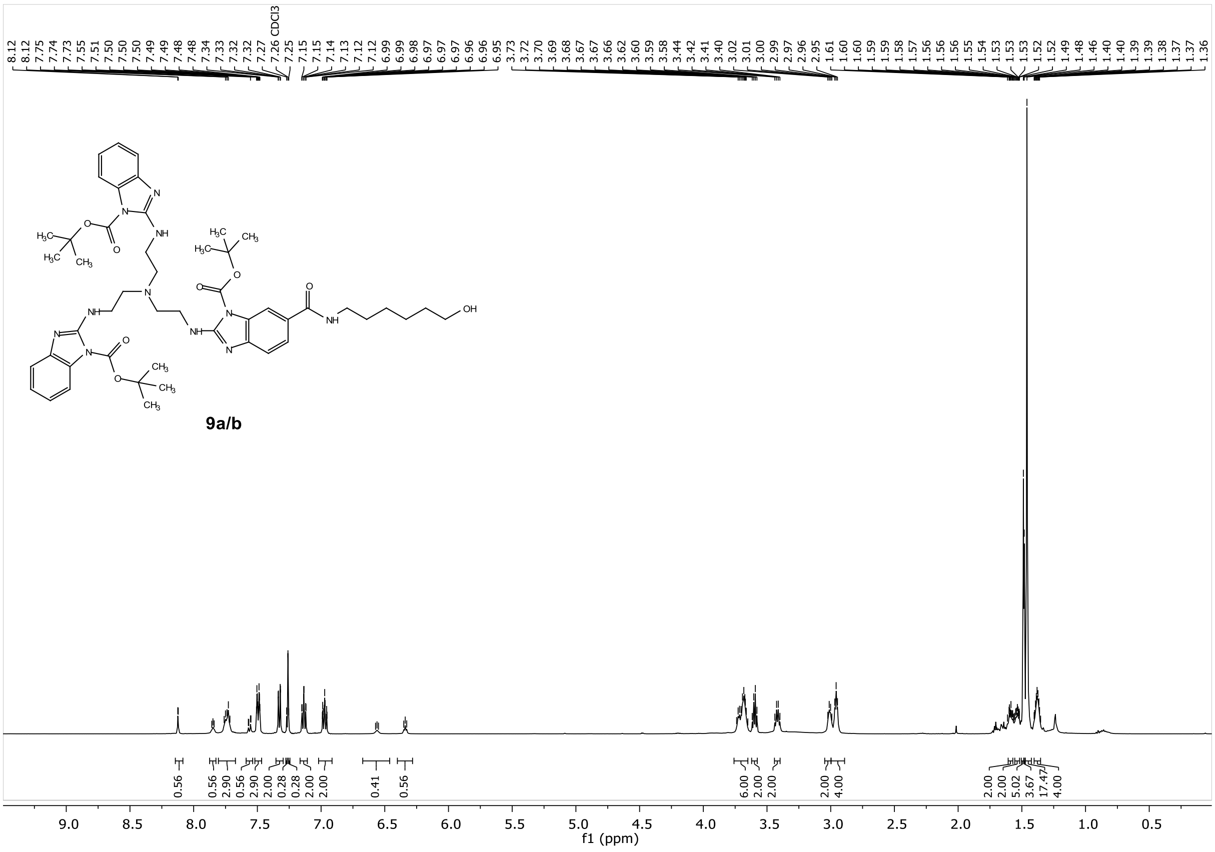
8.12 8.12 7.75 7.74 7.73 7.55 7.51 7.50 7.50 7.49 7.49 7.48 7.34 7.33 7.32 7.32 7.27 7.26 CDCl3 7.25 7.15 7.15 7.14 7.13 7.12 6.99 6.99 6.98 6.97 6.97 6.96 6.96 6.95
3.73 3.72 3.70 3.69 3.68 3.67 3.66 3.62 3.60 3.59 3.58 3.44 3.42 3.41 3.40 3.02 3.01 3.00 2.99 2.97 2.96 2.95
1.61 1.60 1.60 1.59 1.59 1.58 1.57 1.56 1.56 1.56 1.55 1.54 1.53 1.53 1.53 1.52 1.52 1.49 1.48 1.46 1.40 1.39 1.39 1.38 1.37 1.37 1.36
0.56 0.56 2.90 0.56 2.90 2.00 0.28 0.28 2.00 2.00 0.41 0.56
6.00 2.00 2.00 2.00 4.00
2.00 2.00 5.02 3.67 17.47 4.00
9a/b
f1 (ppm)

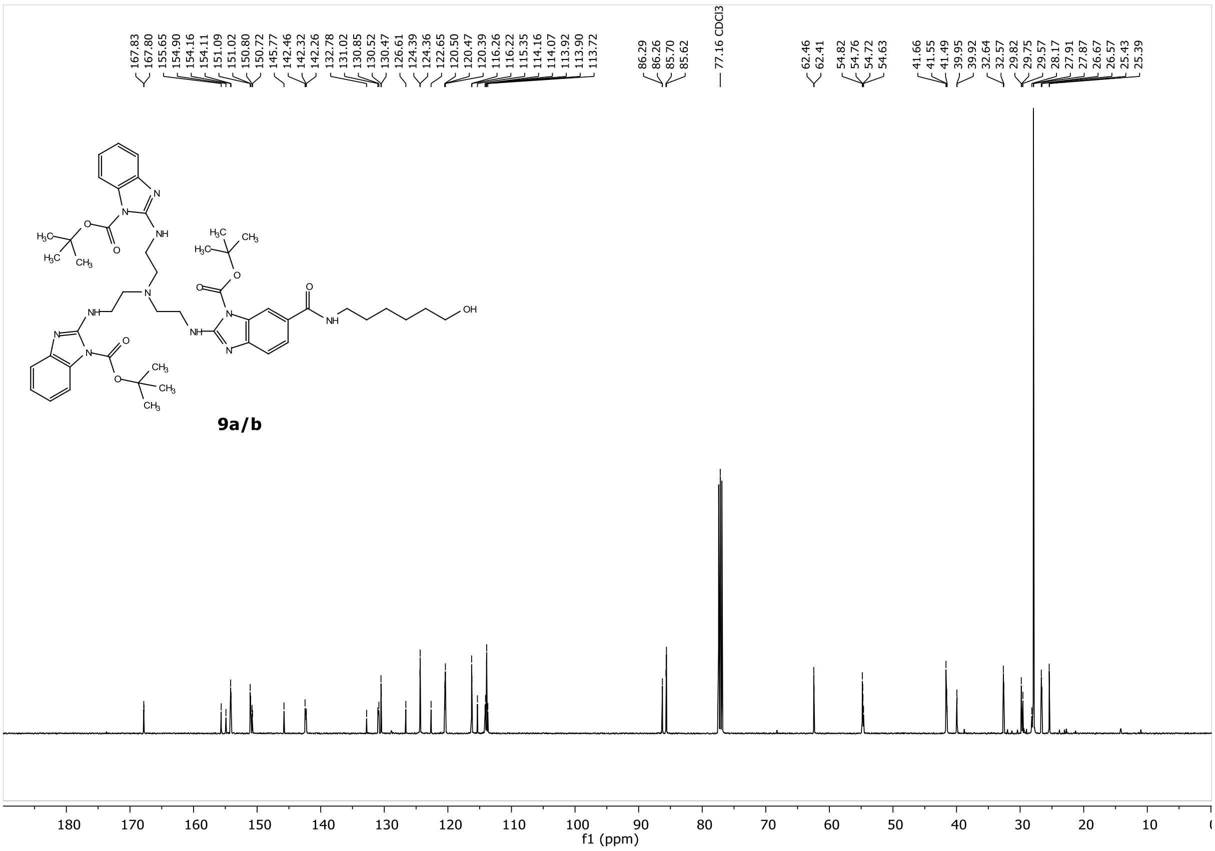
167.83 167.80 155.65 154.90 154.16 154.11 151.09 151.02 150.80 150.72 145.77 142.46 142.32 142.26 132.78 131.02 130.85 130.52 130.47 126.61 124.39 124.36 122.65 120.50 120.47 120.39 116.26 116.22 115.35 114.16 114.07 113.92 113.90 113.72
86.29 86.26 85.70 85.62
77.16 CDCl3
62.46 62.41 54.82 54.76 54.72 54.63
41.66 41.55 41.49 39.95 39.92 32.64 32.57 29.82 29.75 29.57 28.17 27.91 27.87 26.67 26.57 25.43 25.39
9a/b
f1 (ppm)

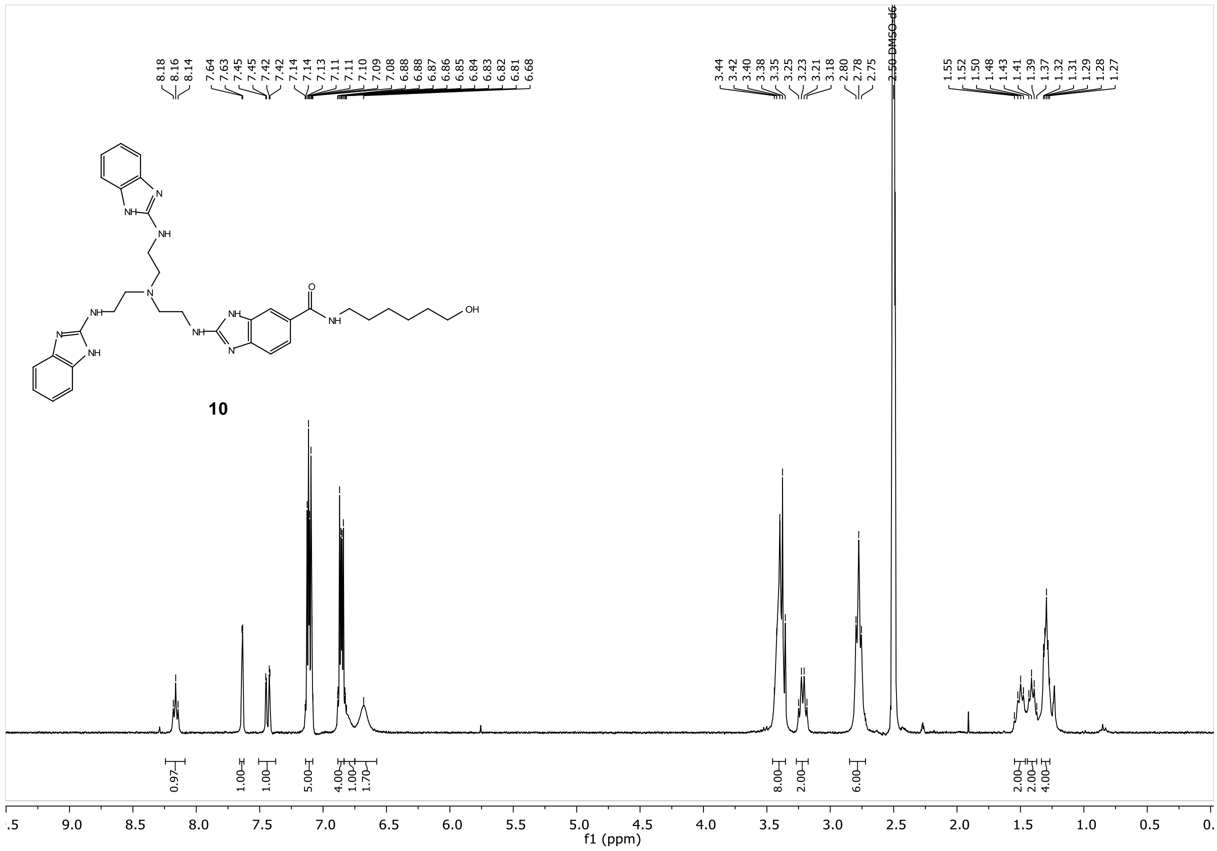
10
f1 (ppm)

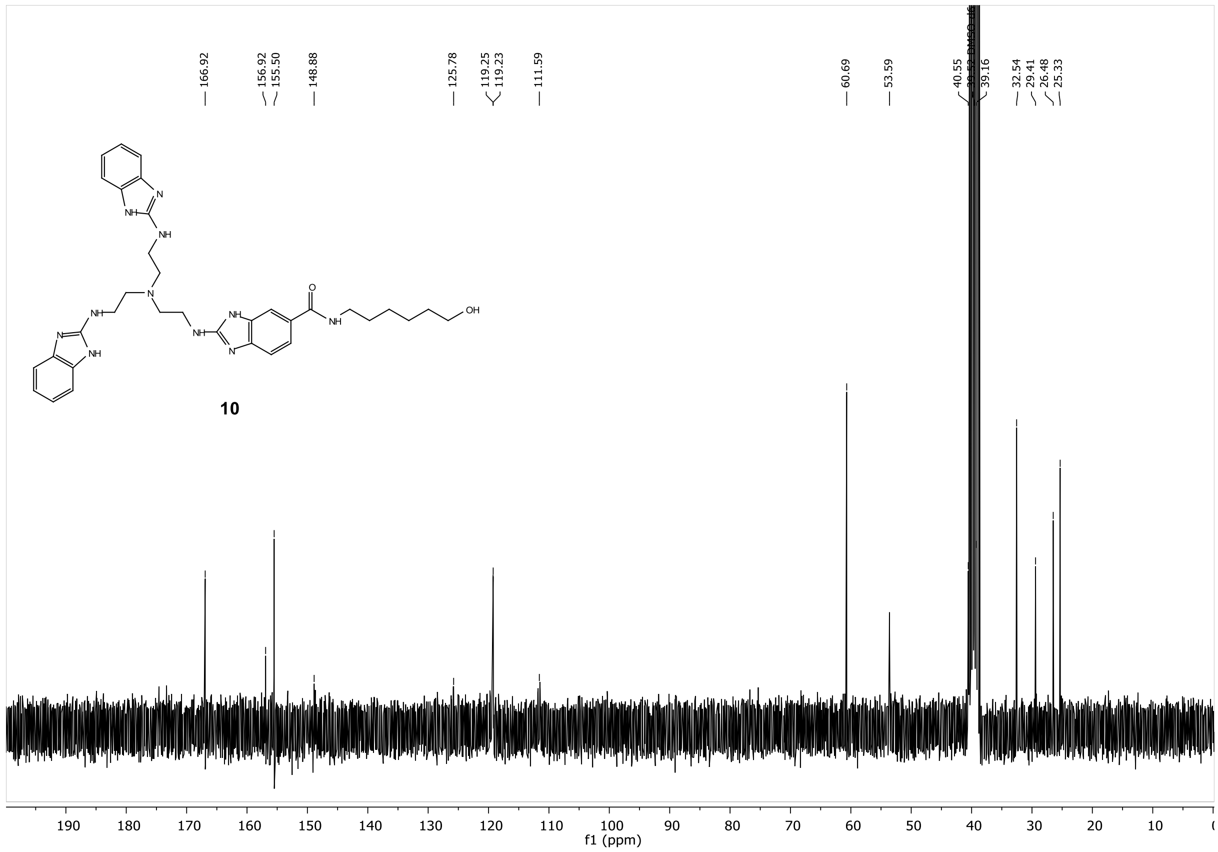
10
f1 (ppm)

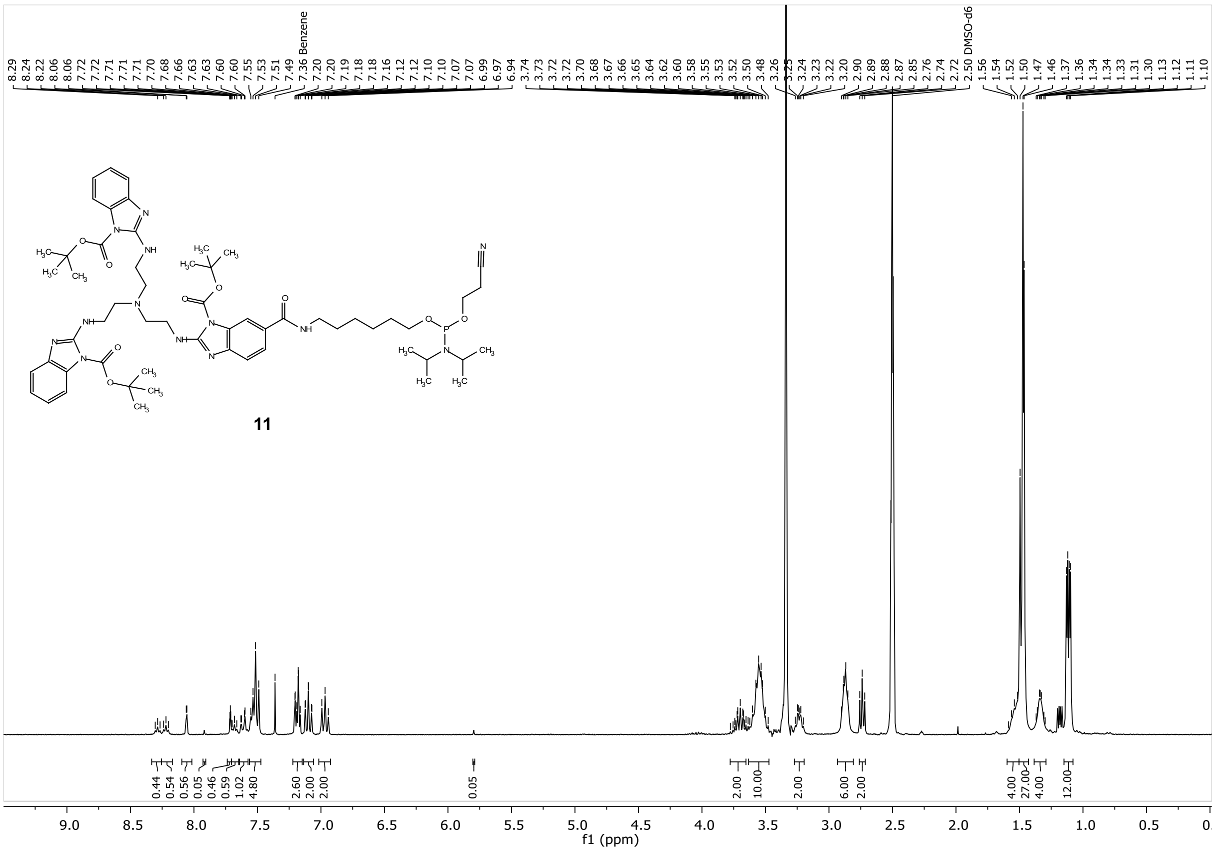
11
f1 (ppm)

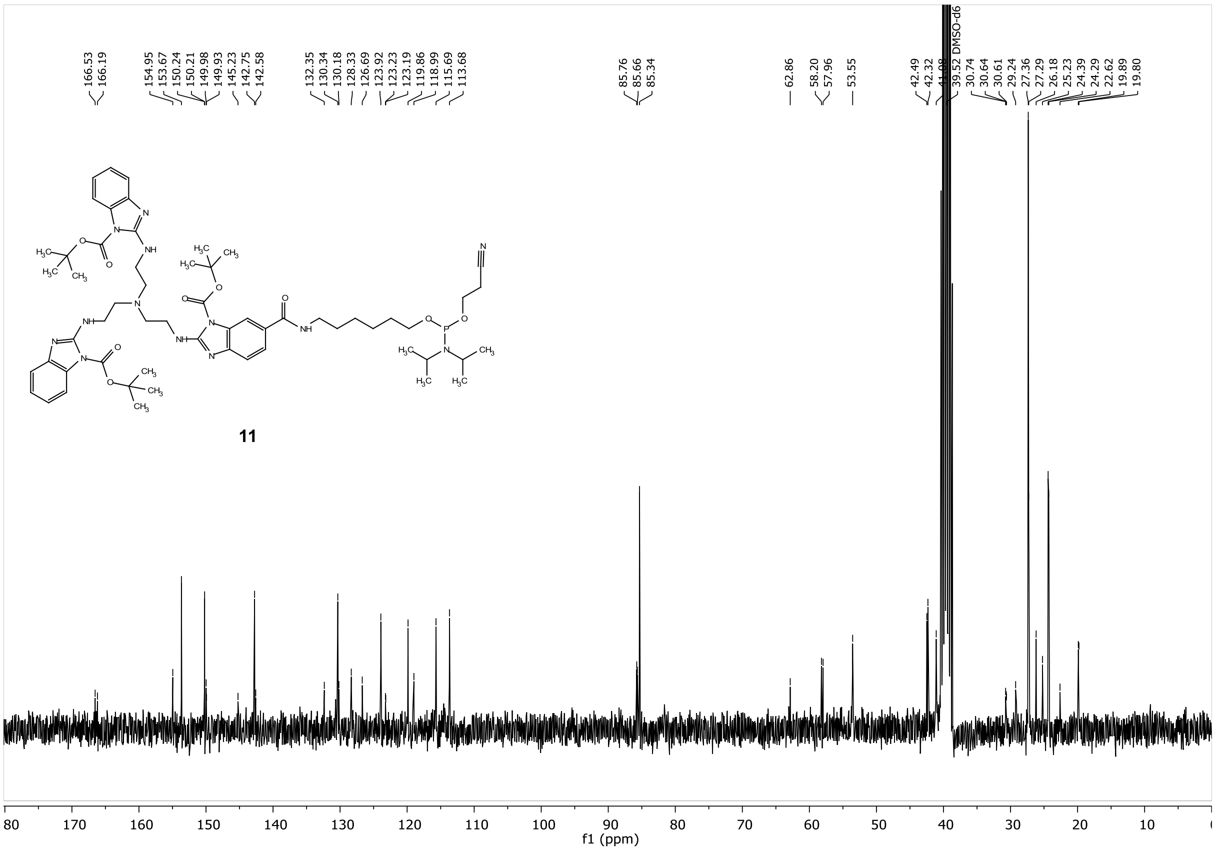
11
f1 (ppm)

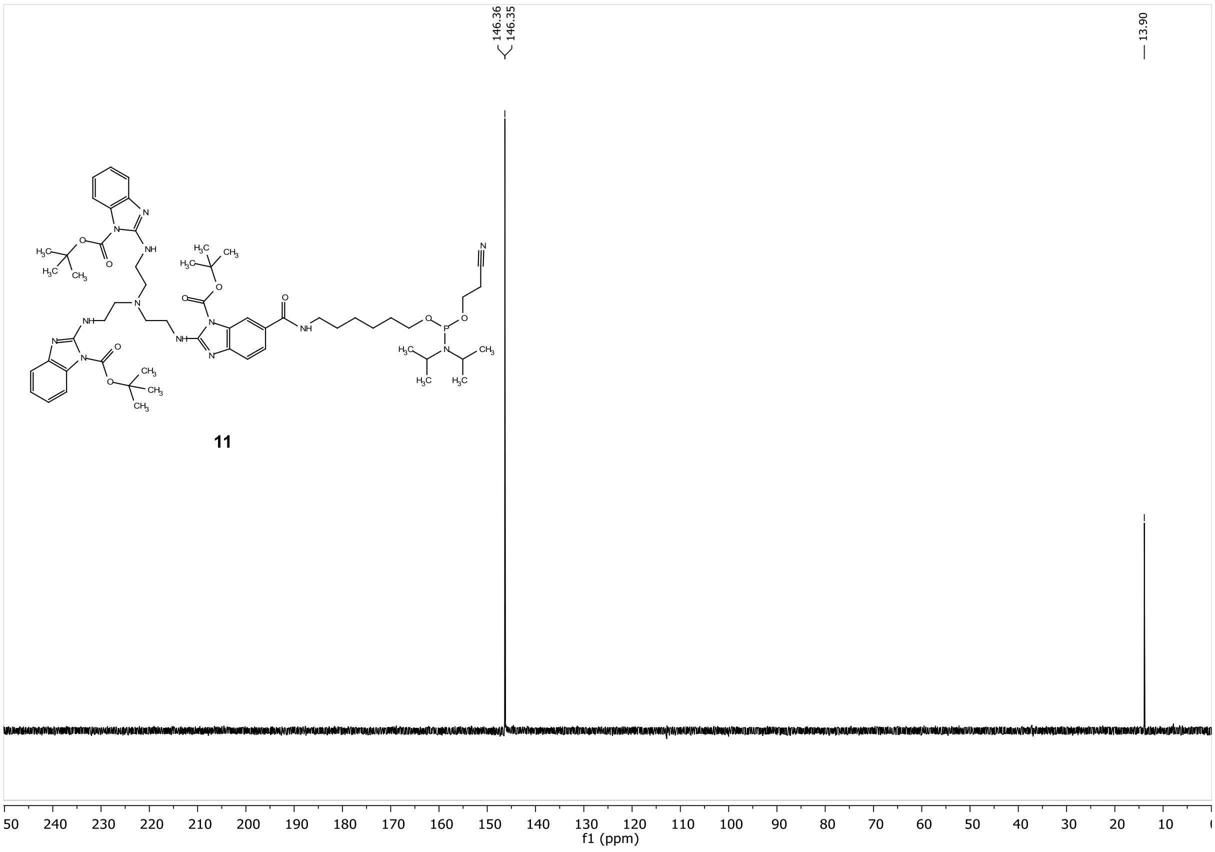
146.36
146.35
13.90
11
f1 (ppm)

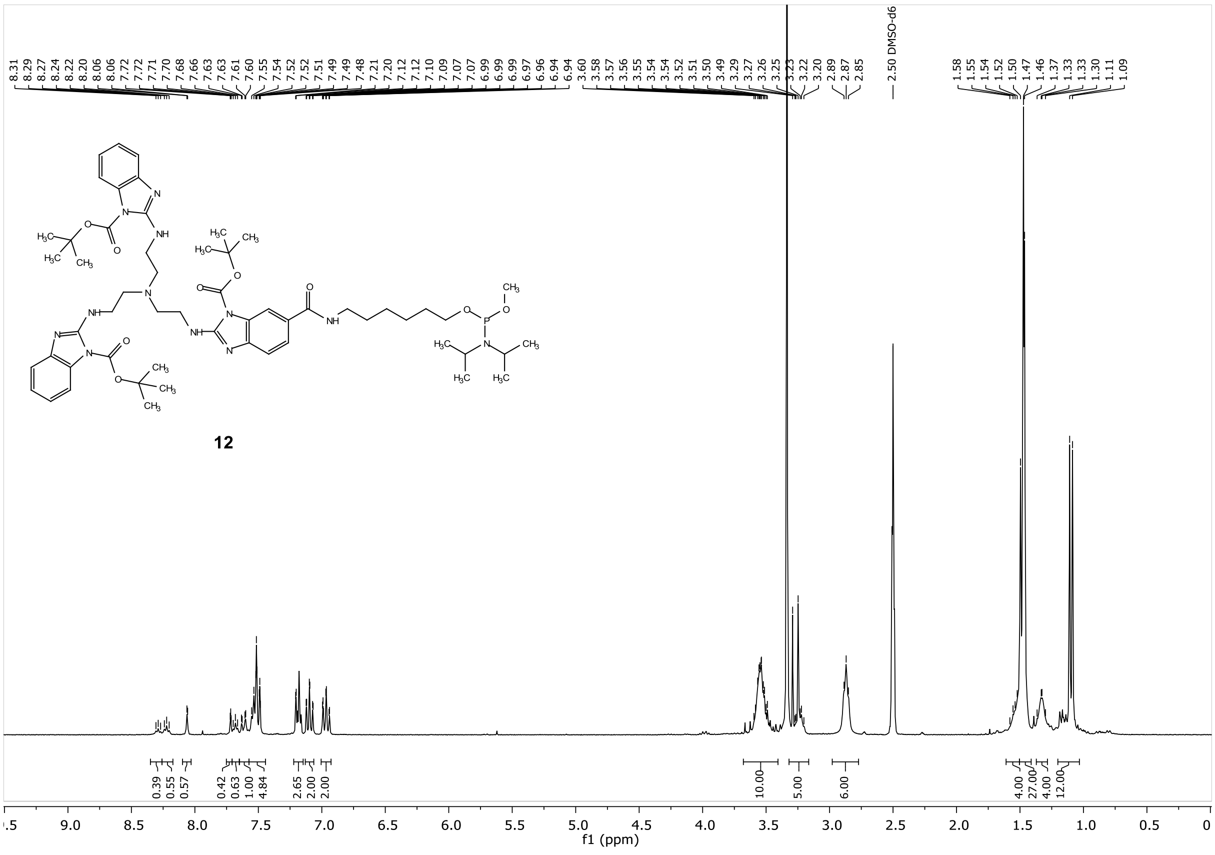
8.31 8.29 8.27 8.24 8.22 8.20 8.06 8.06 7.72 7.72 7.71 7.70 7.68 7.66 7.63 7.63 7.61 7.60 7.55 7.54 7.52 7.52 7.51 7.49 7.48 7.21 7.20 7.12 7.12 7.10 7.09 7.07 7.07 6.99 6.99 6.99 6.97 6.96 6.94 6.94
3.60 3.58 3.57 3.56 3.55 3.54 3.54 3.52 3.51 3.50 3.49 3.29 3.27 3.26 3.25 3.23 3.22 3.20 2.89 2.87 2.85
2.50 DMSO-d6
1.58 1.55 1.54 1.52 1.50 1.47 1.46 1.37 1.33 1.33 1.30 1.11 1.09
12
0.39 0.55 0.57 0.42 0.63 1.00 4.84 2.65 2.00 2.00 10.00 5.00 6.00 4.00 27.00 4.00 12.00
f1 (ppm)

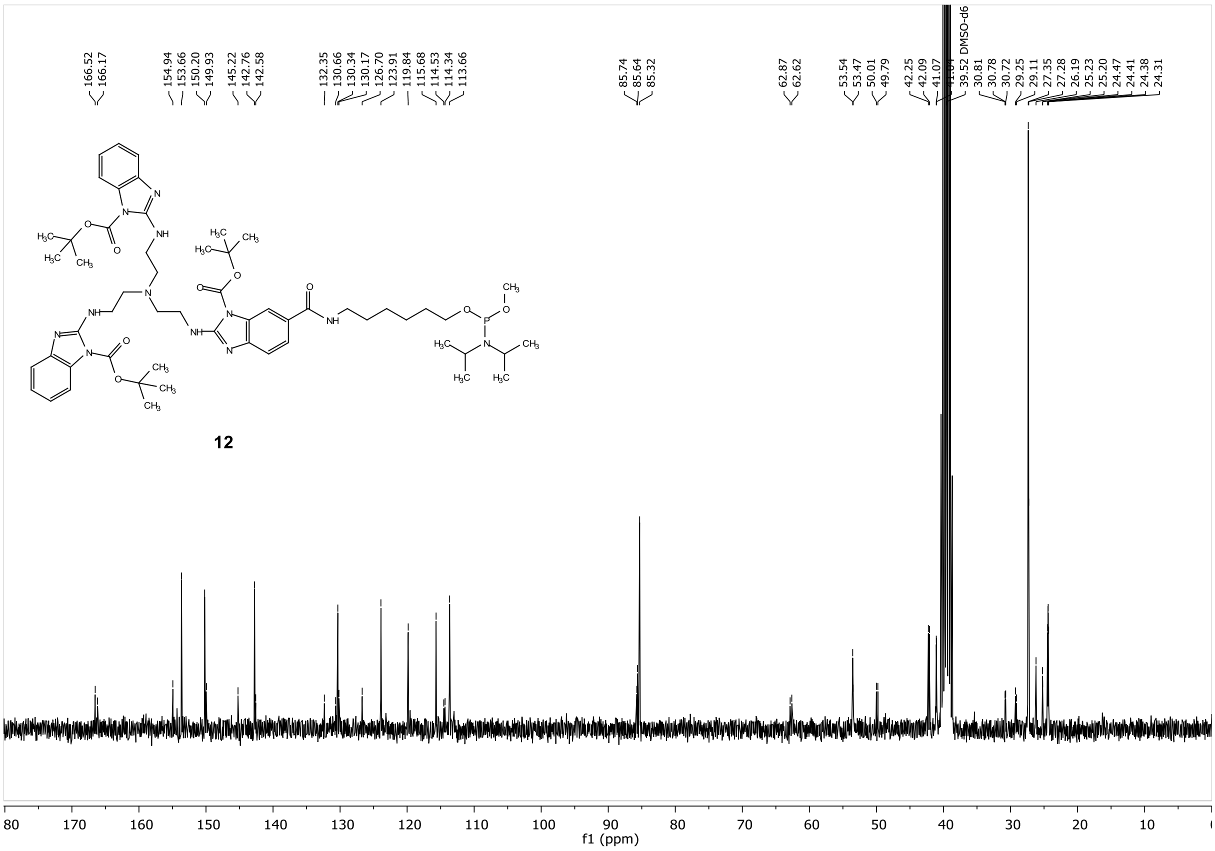
166.52
166.17
154.94
153.66
150.20
149.93
145.22
142.76
142.58
132.35
130.66
130.34
130.17
126.70
123.91
119.84
115.68
114.53
114.34
113.66
85.74
85.64
85.32
62.87
62.62
53.54
53.47
50.01
49.79
42.25
42.09
41.07
39.52 DMSO-d6
30.81
30.78
30.72
29.25
29.11
27.35
27.28
26.19
25.23
25.20
24.47
24.41
24.38
24.31
12
f1 (ppm)

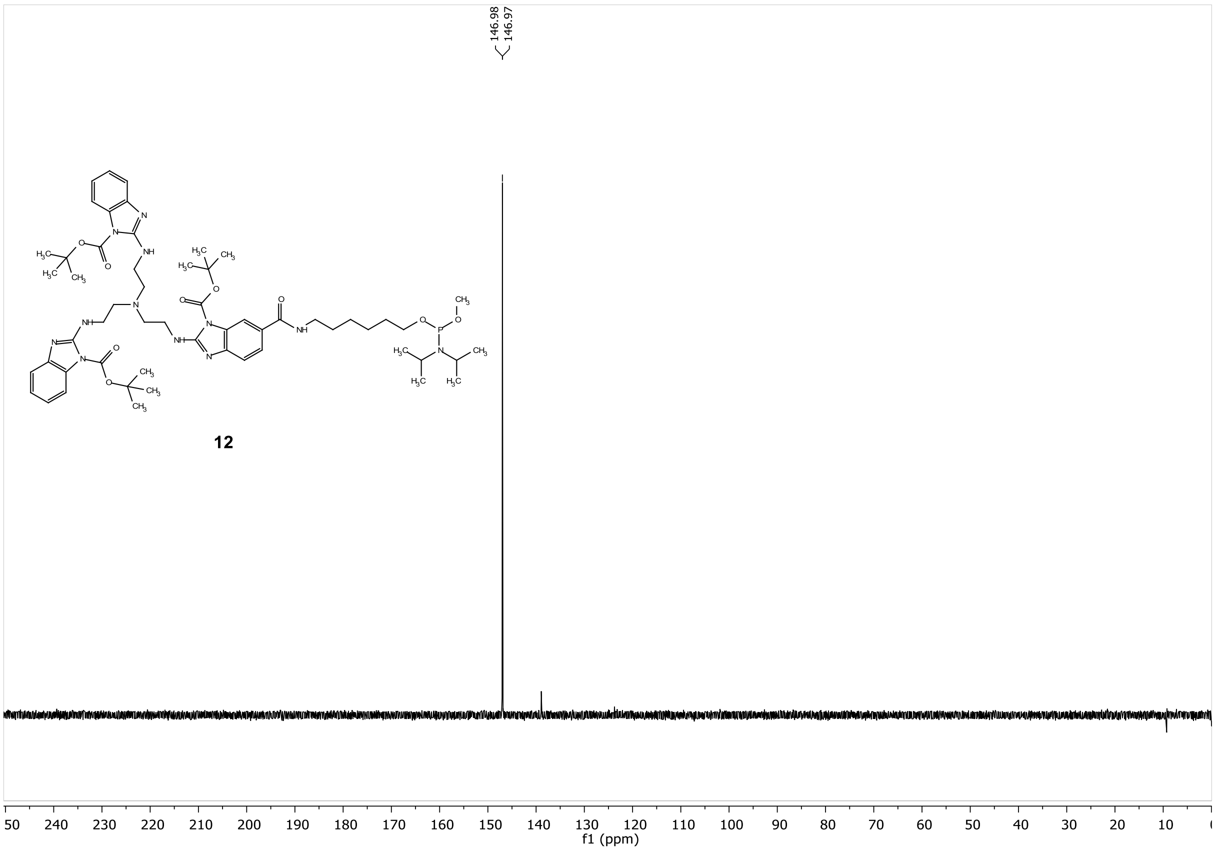
146.98
146.97
12
f1 (ppm)
